# Glycosyltransferase *OsUGT90A1* helps protect the plasma membrane during chilling stress in rice

**DOI:** 10.1093/jxb/eraa025

**Published:** 2020-01-24

**Authors:** Yao Shi, Huy Phan, Yaju Liu, Shouyun Cao, Zhihua Zhang, Chengcai Chu, Michael R Schläppi

**Affiliations:** 1 Department of Biological Sciences, Marquette University, Milwaukee, WI, USA; 2 National Sweet Potato Improvement Center, Sweet Potato Research Institute, Xuzhou, P.R. China; 3 State Key Laboratory of Plant Genomics, Institute of Genetics and Developmental Biology, The Innovative Academy of Seed Design, Chinese Academy of Sciences, Beijing, China; 4 University of Birmingham, UK

**Keywords:** Abiotic stress, antioxidant enzyme activity, cold tolerance, gene knockout, low temperature seedlings survivability, reactive oxygen species, salt tolerance, transgenic rice

## Abstract

Due to its subtropical origins, rice (*Oryza sativa*) is sensitive to low-temperature stress. In this study, we identify LOC_Os04g24110, annotated to encode the UDP-glycosyltransferase enzyme UGT90A1, as a gene associated with the low-temperature seedling survivability (LTSS) quantitative trait locus *qLTSS4-1*. Differences between haplotypes in the control region of *OsUGT90A1* correlate with chilling tolerance phenotypes, and reflect differential expression between tolerant and sensitive accessions rather than differences in protein sequences. Expression of *OsUGT90A1* is initially enhanced by low temperature, and its overexpression helps to maintain membrane integrity during cold stress and promotes leaf growth during stress recovery, which are correlated with reduced levels of reactive oxygen species due to increased activities of antioxidant enzymes. In addition, overexpression of *OsUGT90A1* in Arabidopsis improves freezing survival and tolerance to salt stress, again correlated with enhanced activities of antioxidant enzymes. Overexpression of *OsUGT90A1* in rice decreases root lengths in 3-week-old seedlings while gene-knockout increases the length, indicating that its differential expression may affect phytohormone activities. We conclude that higher *OsUGT90A1* expression in chilling-tolerant accessions helps to maintain cell membrane integrity as an abiotic stress-tolerance mechanism that prepares plants for the resumption of growth and development during subsequent stress recovery.

## Teaser

Glycosyltransferase *OsUGT90A1* helps to protect cellular membranes during chilling and salt stress by enhancing antioxidant enzyme activity to reduce ROS levels, and to balance the stress response with growth.

## Introduction

Rice (*Oryza sativa*) is an important staple crop, feeding almost three billion people worldwide (Fairhurst and Dobermann, 2002; Shelton *et al.*, 2002). Due to its subtropical origins, rice is susceptible to chilling stress (0–15 °C). Rice grown in temperate regions is often exposed to temperatures below 10 °C, and hence yields are severely affected (Xie *et al.*, 2012). Only a few chilling-tolerance genes have been identified using map-based cloning strategies (da Cruz *et al.*, 2013; Zhang *et al.*, 2014); however, because of the large amount of genetic variation within genotyped rice accessions from around the world, numerous cold-tolerance quantitative trait loci (QTL) have been identified through genome-wide association studies (GWAS) (da Cruz *et al.*, 2013; Fujino *et al.*, 2015; Pan *et al.*, 2015; Zhu *et al.*, 2015; Lv *et al.*, 2016; Wang *et al.*, 2016*a*; Shakiba *et al.*, 2017; Schläppi *et al.*, 2017). Such mapping approaches are facilitated by the fact that rice has two major subspecies with considerable genetic variation. The relatively chilling-tolerant *japonica* subspecies is composed of the three subpopulations ‘aromatic’, ‘temperate japonica’, and ‘tropical japonica’, and the chilling-sensitive *indica* subspecies is composed of ‘aus’ and various ‘indica’ subpopulations.

Low-temperature stress negatively affects plant growth and development by altering the physical and chemical properties of the plasma membrane, and by disrupting ion homeostasis and overall cell metabolism (Thomashow, 1999; Ruelland and Zachowski, 2010; Ding *et al.* 2019). Plants utilize various strategies to protect themselves from low-temperature stress. An intact membrane is crucial for effective cold-stress response mechanisms, and the lipid compositions of plasma- and endo-membranes undergo remarkable changes during cold acclimation that help to alleviate the impact of cellular damage induced by low-temperature stress (Uemura *et al.*, 2006; Takahashi *et al.*, 2013; Barrero-Sicilia *et al.*, 2017). However, this protective mechanism is simultaneously challenged by oxidative stress derived from reactive oxygen species (ROS) that are induced by cold temperatures.

ROS such as the superoxide ion (O_2_^•–^) and hydrogen peroxide (H_2_O_2_) are inevitable byproducts of cellular metabolism (Mittler *et al.*, 2011). ROS can be categorized into metabolic and signaling, with the former originating from disruption of cellular metabolism as a consequence of stresses and the latter originating from sensors that receive the stress signals (Mittler and Blumwald, 2010; Mittler *et al.*, 2012). The two groups are produced via different biosynthesis pathways in multiple cellular organelles, such as the plasma membrane, chloroplasts, mitochondria, peroxisomes, and the vacuole (Suzuki *et al.*, 2011; Vaahtera *et al.*, 2014; Gilroy *et al.*, 2016; Mignolet-Spruyt *et al.*, 2016). Excessive ROS can cause substantial molecular, biochemical, and physiological damage through over-oxidation of vital macromolecules such as lipids, amino acids, and DNA (Gill and Tuteja, 2010; Krasensky and Jonak, 2012). To cope with oxidative stress, higher plants have evolved mechanisms that utilize a variety of antioxidants to scavenge ROS. Antioxidants can be classified into enzymatic antioxidants, such as ascorbate peroxidase (APX), catalase (CAT), glutathione peroxidase (GPX), and superoxide dismutase (SOD), and non-enzymatic antioxidants, such as ascorbic acid and glutathione (Mittler *et al.*, 2004). Flavonoids and anthocyanins can also scavenge ROS (Gupta *et al.*, 2010) and the last step of their biosynthesis is catalysed by specific UDP-flavonoid/anthocyanin glycosyltransferases (UFGTs), generating various derivatives with antioxidant activity (Shi and Xie, 2014). The rice genome has 793 putative UDP-glycosyltransferases (UGTs; Cao *et al.*, 2008), some of which are functionally characterized as UFGTs (Ko *et al.*, 2006, 2008; Peng *et al.*, 2017). UFGTs and other UGTs are involved in abiotic stress responses (Sun *et al.*, 2013; Ahrazem *et al.*, 2015; Li *et al.*, 2017, 2018), detoxification of molecules produced by biotic stresses, and glycosylation of phytohormones (Jones and Vogt, 2001; Brazier *et al.*, 2002; Bowles *et al.*, 2005). Phytohormones play essential roles in the regulation of plant growth and development, and in responses to biotic and abiotic stress (Ku *et al.*, 2018). The activity of phytohormones such as auxins, cytokinins, and abscisic acid (ABA) can be regulated by UGT-mediated glycosylation (Jackson *et al.*, 2001; Xu *et al.*, 2002; Hou *et al.*, 2004), which generally serves as an inactivation mechanism for secondary plant metabolites (Jones and Vogt, 2001).

In this study, we show that the rice UGT gene *OsUGT90A1* is associated with the low-temperature seedling survivability (LTSS) QTL *qLTSS4-1* (Schläppi *et al.*, 2017). Overexpression of *OsUGT90A1* helps to protect the plasma membrane against cellular damage induced by abiotic stress, and is correlated with an increase in antioxidant enzyme activity and a reduction in ROS levels during abiotic stress. *OsUGT90A1* has a biphasic expression pattern in rice seedlings exposed to low-temperature stress, most likely because the gene affects developmental processes such as root and shoot growth. A model for *OsUGT90A1* function is proposed and discussed.

## Materials and methods

### Plant materials and growth condition

The USDA Rice Mini-Core (RMC) collection (Li *et al.*, 2010) consists of 203 *Oryza sativa* germplasms categorized into six subpopulations: ‘aromatic’ (six accessions), ‘tropical japonica’ (33), and ‘temperate japonica’ (34) from the *japonica* subspecies, ‘aus’ (38) and ‘indica’ (68) from the *indica* subspecies, and 24 accessions that are an admixture of two or more subpopulations. The RMC collection was used as the main experimental material for both genome-wide association study (GWAS) mapping and phenotyping of chilling tolerance. Two rice reference accessions, ‘temperate japonica’ Nipponbare and ‘aus’ Kasalath, and two Arabidopsis ecotypes, Columbia-0 (Col-0) and Landsberg *erecta*-0 (L*er*-0), were used to generate overexpression (OX) transgenic lines, and the ‘temperate japonica’ accession Zhong Hua 11 (ZH11) was used to generate knockout lines.

Rice seedlings were grown hydroponically in an AR-66L growth chamber (Percival Scientific, Inc.) under ~165 µE photon flux and 12/12 h light/dark conditions at 28/25 °C. Water was replaced by quarter-strength Murashige and Skoog (MS) liquid medium on day 7. All wild-type and transgenic Arabidopsis seeds were surface-sterilized with 10% sodium hypochlorite and germinated on agar-solidified half-strength MS medium in an AR-66L growth chamber under standard growth conditions of 16/8 h light/dark at 22/20 °C and ~165 µE photon flux.

### Selection of a chilling-tolerance candidate gene associated with *qLTSS4-1*

Using online databases, a 1-Mb genomic region upstream and downstream from the small sequence repeat (SSR) marker RM3317 associated with the *qLTSS4-1* locus was scanned for putative cold-tolerance candidate genes as follows. First, the Gene Ontology Consortium (http://www.geneontology.org) and QTARO (Yonemaru *et al.*, 2010) databases were used to assign functions logically connected to the three potential chilling-tolerance gene classes ‘sensing/signal transduction’, ‘transcription factor’, and ‘effector’, or as to assign them as ‘hypothetical’. Second, genes that were predominantly expressed in tissues associated with LTSS, such as ‘seedling’, ‘leaf’, and also ‘root’, were selected from the Rice Expression Profile Database (http://ricexpro.dna.affrc.go.jp). Third, the file ‘final_rice_v7_expression_matrix_48columns.txt’ from the MSU Rice Genome Annotation Project (http://rice.plantbiology.msu.edu/index.shtml; Kawahara *et al.*, 2013) was used to select genes that were cold-induced and differentially expressed between the two reference genomes Nipponbare and 93-11 (‘indica II’), and was supplemented with information from previously published differential gene expression studies done under low-temperature stress (Yun *et al.*, 2010; Zhang *et al.*, 2012; Chawade *et al.*, 2013). Finally, The SNP-Seek finder (http://snp-seek.irri.org; Alexandrov *et al.*, 2015) and RiceVarMap (http://ricevarmap.ncpgr.cn; Zhao *et al.*, 2015) browsers together with RMC re-sequencing data (Wang *et al.*, 2016*b*) were used to select candidate genes with single-nucleotide polymorphisms (SNPs) in the coding and upstream regulatory regions of chilling-tolerant and chilling-sensitive accessions, with special emphasis on SNPs that generated non-synonymous amino acid substitutions.

Taken together, a gene was considered as a probable *qLTSS4-1*-associated gene if it was annotated either as ‘sensing/signal transduction’, ‘transcription factor’, or ‘effector’, if it was expressed predominantly in seedling tissues, if it was differentially expressed between *japonica* and *indica* accessions; if it was cold-temperature regulated; and if it had SNPs in both the upstream regulatory and coding regions, with the latter generating non-synonymous amino acid substitutions. A haplotype map was then generated for these genes using the RiceVarMap browser and RMC re-sequencing data. The genes for which the haplotype region was in linkage disequilibrium were then selected as the most probable candidate genes.

### Cold-temperature stress treatment of rice plants

Rice seeds were germinated and grown as described above. Two-week-old seedlings from the RMC used for GWAS mapping were stressed continuously at 10 °C for 7 d. For transgenic plants, the stress conditions were 2 d continuously at 10 °C for the *OsUGT90A1*-OX Kasalath lines, and 4 d continuously at 4 °C for the *OsUGT90A1*-OX Nipponbare and *osugt90a1*-KO ZH11 lines. All assays related to chilling stress were done after completion of these cold-temperature treatments. See below for details of construction of the transgenic plants.

### Assessment of low-temperature seedling survivability (LTSS) in rice

Seedlings at 2 weeks old were cold-temperature stressed as described above and allowed to recover for 1 week at 28/25 °C day/night cycles with a 12/12 h photoperiod at ~165 µE photon flux. Mean survivability was calculated as %LTSS, which was the number of green and healthy-looking seedlings after 1 week of recovery growth divided by the number of initial healthy-looking seedlings before treatment, expressed as a percentage. Nipponbare and Krasnodarskij 3352 (‘temperate japonica’) were used as chilling-tolerant checks, and Kasalath and Carolino 164 (‘aus’) were used as chilling-sensitive checks.

### Measurement of electrolyte leakage

Immediately after the low-temperature stress treatments, four equally sized tissue sections from the middle part of different leaf blades were collected from the rice seedlings, and four leaves were removed from Arabidopsis seedlings. The leaf samples were washed in ddH_2_O, transferred into screw-cap glass tubes filled with 5 ml of ddH_2_O, and placed on a rotary shaker at 200 rpm for 1 h to release cellular electrolytes from damaged cells. Electrolyte leakage (EL) of each of the four replicate samples was measured twice in separate 120-µl aliquots of the solution using a hand-held LAQUAtwin B-771 conductivity meter (Horiba Scientific, Japan). Care was taken to ensure that the glass tubes were free of ions and that the ddH_2_O used had no significant conductivity. After the initial EL measurement, the tissue samples were incubated in boiling water for 10 min to release the total electrolyte contents of all cells. After cooling to room temperature, the samples were shaken at 200 rpm for 1 h and EL was measured again. Electrolyte leakage for each treatment was then determined as %EL = [(initial EL)/(total EL)] ×100.

### Assessment of tolerance to freezing stress in Arabidopsis

Col-0 and the L*er*-0 wild-type and *OsUGT90A1*-OX transgenic lines were subjected to freezing stress using a refrigerated circulating bath (Polyscience, Niles, IL, USA). Individual seedlings at 2 weeks old were removed from agar plates and transferred onto a stack of pre-wetted Light-Duty Tissue Wipes (VWR, Radnor, PA, USA) in 50-ml conical tubes and covered with pre-wetted Tissue Wipes to maintain humidity. The tubes were incubated in the circulating bath at –2 °C for 15 min followed by addition of ~50 µl of ddH_2_O ice chips to promote ice nucleation in the plant tissues. After further incubation at –2 °C for 1 h, the seedlings were allowed to thaw inside the tubes overnight in the dark at 4 °C. The thawed plant tissues were then subjected to different phenotypic assays.

For assessment of whole-plant survival, tubes containing 2-week-old seedlings were placed in the circulating bath as described above, and after 1 h at –2 °C the temperature in the bath was reduced at a rate of –2 °C h^–1^. Tubes were taken out after 1 h (i.e. –2 °C), and when the temperature reached –4 °C and –6 °C. The seedlings were thawed overnight in the dark at 4 °C and were then transferred to pre-wetted filter papers and placed for 1 week under the standard conditions in the growth chamber described see above.

### Assessment of tolerance to salt stress in Arabidopsis

Salt germination assays were done as described previously (Xu *et al.*, 2011). One hundred surface-sterilized seeds of the L*er*-0 wild-type and of two of the *OsUGT90A1-*OX homozygous transgenic lines were positioned in a grid onto agar-solidified half-strength MS medium supplemented with various concentrations of NaCl (0, 50, 100, 150, and 200 mM) and germinated for 2 weeks under standard growth conditions. Each plate had 33–35 seeds of each line, and the position of seeds from each line was different on each of the three plates used to account for potential positional effects. Germination was defined as protrusion of the radicle and was assessed each day during the 2-week incubation period. For each line it was expressed as a percentage for each replicate plate: %germination = [(number of germinated seeds)/(total number of seeds)] × 100. Mean germination percentages were calculated from replicates.

### Cloning of rice genomic DNA and plant transformation

*OsUGT90A1* was PCR-amplified from genomic DNA of the accessions Krasnodarskij 3352 and Carolino 164. A genomic region of ~500 bp upstream from the coding region was also PCR-amplified from these accessions (for all primers see Supplementary Table S1 at *JXB* online). PCR amplifications were done using Invitrogen Platinum SuperFi DNA polymerase (ThermoFisher Scientific), with the addition of 0.5 µl of ExTaq DNA polymerase (Takara) to add adenines to the 3´-ends of the products, which were ligated into the pGEM-T vector (Promega) and sequenced. To generate the overexpression (OX) constructs, *OsUGT90A1* was ligated into the *Bam*HI and *Eco*RI sites between the strong MAC promoter and mannopine synthase terminator within the binary vector pPZP211. To generate the *OsUGT90A1* promoter::LUC (luciferase) reporter gene constructs, the 500-bp genomic sequence was ligated into the *Kpn*I and *Bam*HI sites in front of the LUC reporter gene within the binary vector pPZP211. To generate OsUGT90A1::eGFP (enhanced green fluorescent protein) fusion protein constructs, genomic DNA was amplified and ligated into the *Xho*I and *Bgl*II sites of pA7-eGFP. The construct was removed from pA7-eGFP and ligated into the *Hind*III and *Eco*RI sites of the binary vector pPZP211. Binary vectors were introduced into *Agrobacterium tumefaciens* strain ABI1 and used for plant transformation.

For Arabidopsis transformation, the floral dip method was used (Clough and Bent, 1998). For rice transformation, embryogenic callus derived from scutellum tissue of the rice reference lines Nipponbare and Kasalath were co-cultivated with *A. tumefaciens* ABI1 and transgenic plants were regenerated as described previously (Toki *et al.*, 2006).

To generate *OsUGT90A1*-knockout (-KO) lines in rice, the GGCGACGACATGGTCCGGGA sequence was inserted into the pRGRB31 vector as the sgRNA target for Cas9 cleavage of *OsUGT90A1* and introduced into *Agrobacterium* strain AGL1. As described previously (Ma and Liu, 2015), the construct was used for *Agrobacterium*-mediated transformation of embryogenic callus derived from scutellum tissue of the accession ZH11. The target gene was PCR-amplified in regenerated plants and two KO lines were confirmed by sequencing.

### Gene expression analysis

Quantitative reverse-transcription PCR (qPCR) analyses were done to measure the relative abundance of mRNA in rice and Arabidopsis plants. Different rice tissues or whole Arabidopsis plants were collected immediately after the various treatments and total RNA was extracted using Invitrogen Trizol reagent (ThermoFisher). First-strand cDNA was synthesized using M-MLV reverse-transcriptase (Promega), and 2× universal SYBR green supermix (BioRad) was used to perform the qPCR reaction in a CFX96 Touch™ Real-Time PCR Detection System using the following conditions: 95 °C for 5 min, followed by 40 cycles of 95 °C for 15 s; 55 °C for 30 s; and 72 °C for 1 min. The *OsUGT90A1* and *ubiquitin* primers are listed in Supplementary Table S1.

### Visualization of *in vivo* luciferase activity and transient transfections assays

Visualization of firefly luciferase (fLUC) activity in live plant tissues was done as described previously (Ishitani *et al.*, 1997). Two-week-old Arabidopsis Col-0 seedlings homozygous for the 500-bp *OsUGT90A1*-promoter::LUC reporter gene construct were incubated in the dark at either room temperature or 4 °C for 3 h, and then seedlings were evenly sprayed three times with a solution of 1 mM luciferin (GoldBio, St. Louis, MO, USA) in 0.01% Triton X-100 and incubated for 5 min in the dark. The resulting bioluminescence signal was recorded in complete darkness using an AutoChemi Darkroom system (UVP, Upland, CA, USA) with an acquisition time of 10 min. The UVP software was used to visualize *in vivo* fLUC activity in transgenic plants, and area density analysis was used to quantify the activity.

To determine fLUC activity in transient transfection assays, rice protoplasts made from young seedling of the 93-11 accession were used, as described previously (Bart *et al.*, 2006; Liu *et al.*, 2018). pPZP211 plasmids containing different 500-bp *OsUGT90A1*-promoter::LUC reporter gene constructs were co-transfected into protoplasts with the pGreenII 0800-LUC vector containing the *Renilla LUC* gene (rLUC) to normalize transfection efficiencies. Protoplasts were incubated in the dark at 28 °C for 12 h, collected by centrifugation at 450 *g* for 3 min, and immediately used for LUC assays. Quantification was done using a dual-luciferase reporter assay kit (Promega). Five independent transformations were done for each construct, and relative LUC activity was calculated as the ratio of fLUC to rLUC.

### Determination of total content of phenolics and anthocyanins in rice

Phenolic and anthocyanin compounds were isolated from 100 mg of 2-week-old leaf tissues using 1 ml of 70:30:1 methanol:water:trifluoroacetic acid extraction buffer and incubated in the dark at room temperature for at least 1 h. After centrifugation (16 000 *g* for 20 min), the supernatant was filtered through a 0.2-µm PTFE membrane using a 13-mm syringe. The total anthocyanin concentration was calculated as [(OD_520_–OD_700_) × MW × DF × 10^3^]/(e × L) where MW is the molecular weight (449.2 g mol^–1^ for cyanidin-3-glucoside, Cyd-3-glu), DF is the dilution factor, L is the pathlength (1 cm), e is the molar extinction coefficient (26 900 mol^–1^ cm^–1^ for Cyd-3-glu), and 10^3^ is the factor for conversion from g to mg. Extraction buffer alone was used as the negative control.

### HPLC-MS analysis of the composition of total phenolics/anthocyanins

The mixture of total phenolics/anthocyanins in extraction buffer was vacuum-dried using a Savant UVS400 Universal Vacuum System (ThermoFisher) and resuspended in 500 μl of pure ddH_2_O. The solvents were H_2_O (A) and CH_3_CN (B) and the gradient elution program was as follows (v/v A in B): start, 10%; 0–30 min, 10–25%; 30–50 min, 25–45%; 50–55 min, 45–100%; 55–60 min, 100%. A 3-μm, 75 × 4.6 mm Gemini 3u C18 110a column was used (Phenomenex, Torrance, CA, USA) with an operating temperature of 40 °C and a flow rate of 1 ml min^–1^. A Shimadzu LC-MS-2020 system was used for mass spectrometry.

### Determination of ROS content and antioxidant activity

Staining with Nitro Blue Tetrazolium (NBT) was used to visualize the O_2_^•–^. Tissues of rice and Arabidopsis were collected from 2-week-old plants before and after chilling stress and were incubated overnight in the dark at room temperature in a solution containing 0.2% NBT (Alfa Aesar, Haverhill, MA, USA) dissolved in 50 mM sodium phosphate buffer (pH 7.5). To remove chlorophyll, the tissues were incubated in 100% ethanol at ~75–80 °C for 4 h before images were taken.

For the reduction assays, 25 μl of undiluted total phenolic/anthocyanin extracts were mixed with 200 μl of 0.1 mM 2,2-diphenyl-1-picrylhydrazyl (DPPH; Sigma) solution and incubated at room temperature for 30 min on a shaker. For the blank controls, 25 μl of the phenolic/anthocyanin extraction buffer were used. Absorbance at 517 mm (OD_517_) was monitored using a spectrophotometer and antioxidant activity was calculated as: {[(OD_517_ blank – OD_517_ sample)/OD_517_ blank] × 100}/total anthocyanin content in mg.

### Propidium iodide staining

Tissue samples of rice or Arabidopsis were submerged for at least 3 h in a solution of 10 μg ml^–1^ propidium iodide (PI; Sigma). The fluorescent signal of PI was visualized using a confocal laser-scanning microscope (Nikon A1R system), with 543 nm excitation and 615 nm emission signals.

### Determination of catalase and peroxidase enzyme activities

Tissue samples (100 mg) of rice or Arabidopsis were extracted with 800 µl of phosphate buffer (pH 7.0) to isolate total proteins. After centrifugation (16 000 *g* for 5 min), the supernatant was diluted 1:1 (v:v) with phosphate buffer. Total protein concentration was determined using Bradford reagent (Fisher Scientific). For the catalase (CAT) assays, discs of filter paper (diameter 8 mm) were soaked for 5 s in protein extract that had been diluted 5-fold, after which the edges of the discs were blotted for 5 s onto a paper towel to absorb excessive liquid. Each disc was then immediately placed at the bottom of a 50-ml glass beaker containing 30 ml of a freshly made 1% H_2_O_2_ solution. The time taken for the disc to reach the surface of the solution was recorded (30 mm distance). CAT activity was calculated as mm s^–1^ mg^–1^ total protein content. The peroxidase assays were done using a QuantiChrom^TM^ kit (BioAssay Systems, Hayward, CA, USA) according to the manufacturer’s instructions, using 10 µl of 20-fold diluted protein extract for each reading.

### Subcellular localization of GFP

Subcellular localization of OsUGT90A1 proteins was determined in protoplasts of ‘indica II’ 93-11 using eGFP fluorescence as described previously (Liu *et al.*, 2018). pPZP211 plasmids containing different *OsUGT90A1::eGFP* fusion constructs were transiently transfected into rice protoplasts using the polyethylene glycol method and incubated in the dark at 28 °C for 12 h. eGFP fluorescence was recorded using a confocal laser-scanning TCS SP8 microscope (Leica). To determine potential co-localization with the plasma membrane, FM^TM^ 4–64 dye [*N*-(3-triethylammoniumpropyl)-4-(6-(4-(diethylamino) phenyl) hexatrienyl) pyridinum dibromide; ThermoFisher] was added as a plasma membrane marker. Internal membranes such as the tonoplast were not stained by FM^TM^ 4–64. Chlorophyll autofluorescence was visualized using 480–515 nm excitation and 650–750 nm detection wavelengths.

## Results

### Identification of *LOC_Os04g24110* as a chilling-tolerance candidate gene associated with *qLTSS4-1*

We previously identified a novel *LOW TEMPERATURE SEEDLING SURVIVABILITY* (*LTSS*) QTL, *qLTSS4-1*, using the RMC collection and a GWAS mapping approach (Schläppi *et al.*, 2017). To identify *qLTSS4-1* candidates, we screened non-transposon genes near *qLTSS4-1*. *LOC_Os04g24110*, annotated to encode the putative anthocyanin 3-O-beta-UDP-glycosyltransferase (UGT) enzyme UGT90A1, was identified as the most probable candidate gene that met all the screening criteria (see Methods), namely temporal and spatial expression patterns, differential expression between chilling-tolerant Nipponbare (‘temperate japonica’) and chilling-sensitive 93-11 (‘indica II’), evidence for low-temperature regulation, and numerous single-nucleotide polymorphisms (SNPs) in both the promoter and coding regions between the three rice reference genomes Nipponbare, 93-11, and Kasalath (‘aus’, *indica*).

**Fig. 1.**
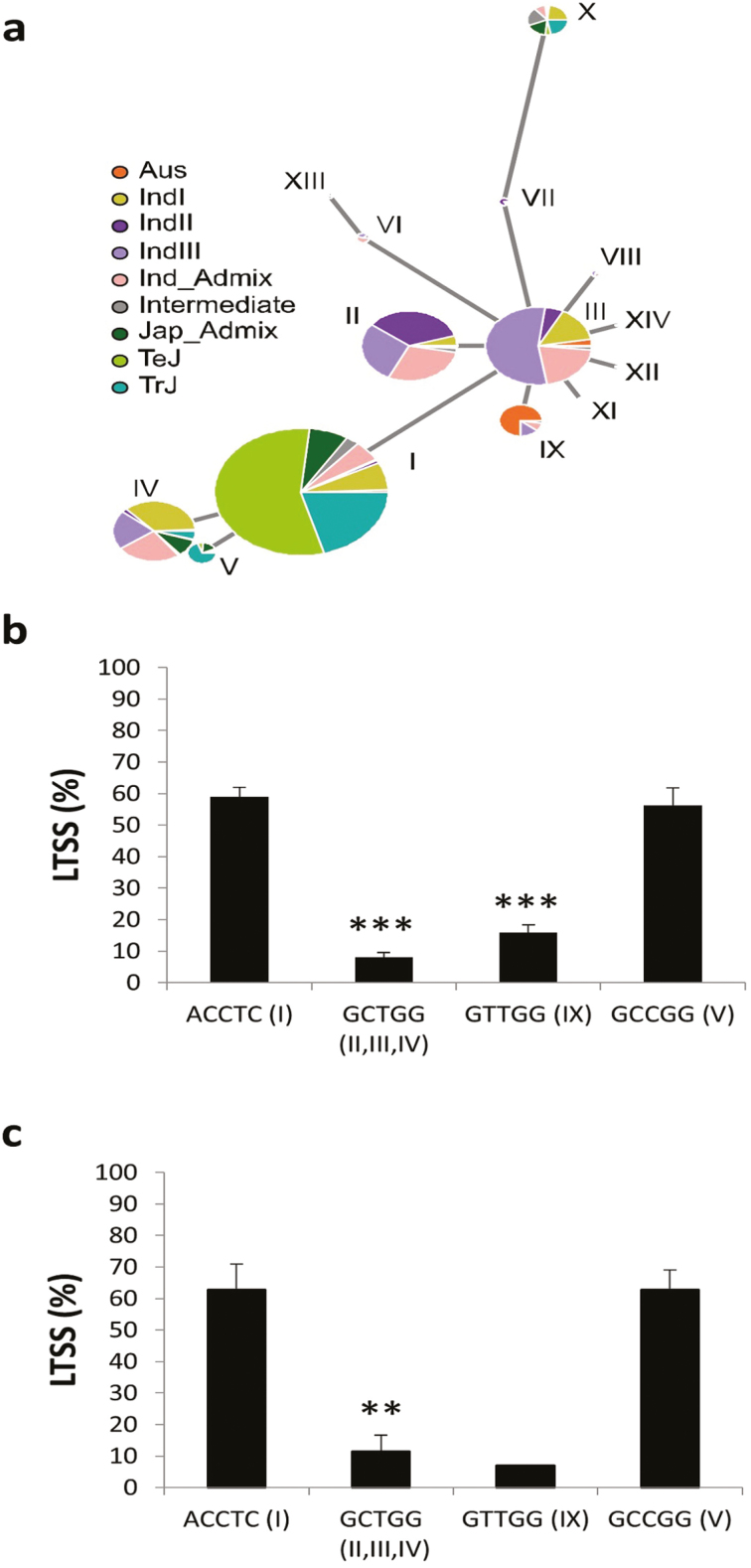
Correlation of *LOC_Os04g24110* haplotypes with percent low-temperature seedling survival (LTSS) in different subpopulations of the Rice Mini-Core (RMC) collection. (a) Haplotype analysis based on 37 single-nucleotide polymorphisms (SNPs) of a 2000-bp genomic DNA fragment spanning *LOC_Os04g24110* using RiceVarMap data from a population of 4402 accessions. Haplotypes (Roman numerals) are representative for the subpopulations ‘temperate japonica’ (TeJ), ‘tropical japonica’ (TrJ), ‘various indica’ (Ind), ‘aus’ (Aus), ‘indica admixtures’ (Ind_Admix), ‘japonica admixtures’ (Jap_Admix), and various intra-subspecies ‘admixtures’ (Intermediate). (b) Haplotype–LTSS correlation analysis based on five SNPs using all 203 *Oryza sativa* accessions of the RMC. Accessions with the ACCTC haplotype belong to the 37-SNP-based haplotype I; accessions with GCTGG belong to haplotypes II, III, and IV; accessions with GTTCC belong to haplotype IX; and accessions with GCCGG belong to haplotype V. (c) Haplotype–LTSS correlation analysis based on five SNPs using only the 24 ‘admixture’ accessions of the RMC. RMC accessions at 2 weeks old were exposed to 10 °C for 7 d and allowed to recover at warm temperatures for 1 week (28/25 °C day/night, 12-h photoperiod), after which LTSS was determined. Data are means (±SE), *n*=5. Significant differences compared to the ACTCC accessions were determined using two-tailed Student’s *t*-tests: **P<10^–4^; ***P<10^–20^.

A haplotype analysis of 2000 bp of genomic DNA of *LOC_Os04g42110*, including 500 bp upstream of the coding region, identified 37 SNPs and 14 haplotypes of which seven were major ones (I, II, III, IV, V, IX, and X) found in at least 100 out of the population of 4402 accessions analysed (Fig. 1a, Supplementary Fig. S1). I and V were predominantly found in accessions of the *japonica* subspecies, including ‘japonica admixtures’, with frequencies of 83.77% and 93.63%, respectively. In contrast, II, III, IV, and IX were predominantly found in accessions of the *indica* subspecies, including ‘indica/aus admix’, with frequencies of 96.90% for II, 98.41% for III, 84.96% for IV, and 97.39% for IX. Haplotype X was equally distributed between accessions of the *japonica* and *indica* subspecies (43.94% and 35.99%, respectively). Haplotypes II, III, and IX were phylogenetically similar as they clustered together, while IV clustered with I and V.

By interrogating the RMC re-sequencing data (Wang *et al.*, 2016b), we reduced the haplotype complexity from seven to four groups by using five SNPs, the first three of which corresponded to the last three SNPs in the 5´ upstream region whilst the fourth and fifth corresponded to the first and last SNPs in the coding region (Supplementary Fig. S1). These four haplotypes were found in different RMC subpopulations as follows. ACCTC (haplotype I; Fig. 1a) was found in 34 out of 34 (100%) ‘temperate japonica’ accessions, and in 28 out of 33 (85%) ‘tropical japonica’ accessions; GCTGG (haplotypes II, III, IV) was found in 51 out of 68 (75%) ‘indica’ accessions; GTTGG (haplotype IX) was found in 29 out of 38 (76%) ‘aus’ accessions, and in 1 out of 68 (1.5%) ‘indica’ accessions; and GCCGG (haplotype V) was exclusively found in 5 out of 33 (15%) ‘tropical japonica’ accessions. A correlation analysis between all RMC accessions having either of these haplotypes and their respective LTSS scores (Fig. 1b) indicated that those containing GCTGG and GTTGG had, as a group, significantly lower scores at *P*-values of 2.59×10^–32^ and 1.55×10^–21^, respectively, than those containing ACTCC, while there was no difference between those containing the ACTCC and GTTGG haplotypes (*P*=0.6595). When all 67 *japonica* accessions were removed from the analysis, ‘indica’, ‘aus’, and ‘admix’ accessions with the ACCTC haplotype had significantly higher LTSS scores than those containing GCTGG (*P*=1.38×10^–5^) and GTTGG (*P*=0.0017). An analysis using only the 24 RMC ‘admix’ accessions showed that 11 and eight accessions contained the ACCTC and GCTGG haplotypes, respectively, and their LTSS score distributions were significantly different with a *P*-value of 6.80×10^–5^ (or *P*=5.02×10^–5^ if the lone accession with the GTTGG sequence was included) while there was no difference between accessions containing the ACCTC and GCCGG haplotypes (*P*=0.9906; Fig. 1c). These results indicated that differences in either gene expression and/or protein structure of *LOC_Os04g24110* (=*OsUGT90A1*) correlated with the observed differences in chilling tolerance between the *japonica* and *indica* accessions of the RMC collection.

### Correlation of *OsUGT90A1* expression with chilling-tolerance phenotypes

**Fig. 2.**
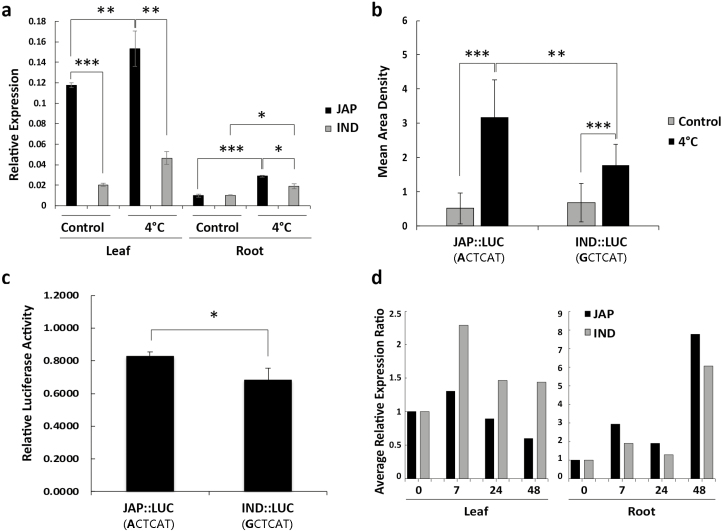
mRNA abundance of *OsUGT90A1* in different rice tissues and in transgenic Arabidopsis plants at warm (control) and cold temperatures. (a) Relative expression of *OsUGT90A1* in leaves and roots at control temperatures (28/25 °C day/night, 12-h photoperiod) and after continuous exposure to 4 °C for 7 h, as determined by qPCR analysis. (b) Quantification of real-time firefly luciferase (LUC) intensity in whole plants of homozygous Arabidopsis lines transformed with a 500-bp *OsUGT90A1*-promoter::LUC construct before (control) and after chilling treatment at 4 °C for 3 h. (c) Normalized LUC)bioluminescent activity at 28 °C in rice protoplast transiently transfected with plasmids containing a 500-bp *OsUGT90A1*-promoter::LUC reporter gene. (d) Time-series analysis of *OsUGT90A1* expression in leaves and roots during exposure to 4 °C, as determined by qPCR analysis. Relative expression levels at 7, 24, and 48 h were normalized to the level of the warm-temperature control (time 0, set as 1). JAP, *japonica* accession Krasnodarskij 3352 (haplotype I; Fig. 1a); IND: *indica* accession Carolino 164 (haplotype IX; Fig. 1a). JAP::LUC, a 500-bp *OsUGT90A1* promoter fragment from Krasnodarskij 3352 fused to LUC; IND::LUC, a 500-bp *OsUGT90A1* promoter fragment from Carolino 164 fused to LUC. Data are means (±SD), *n*=3 (a, b, d), *n*=10 (c). Significant differences between means are indicated and were determined using two-tailed Student’s *t*-tests: *P<0.05; **P<0.01; ***P<0.001.

Previous metadata analyses have indicated that *OsUGT90A1* has higher expression in chilling-tolerant Nipponbare (‘temperate japonica’) than in chilling-sensitive 93-11 (‘indica II’) and that it is differentially expressed during exposure to low temperature (Zhang *et al.*, 2012). To confirm these expression profiles for RMC cultivars, we selected Krasnodarskij 3352 (ACCTC haplotype I, *japonica*) and Carolino 164 (GTTGG haplotype IX, *indica*) as representatives for the two subspecies, because they are known to have consistently high and low cold-tolerance scores, respectively (Schläppi *et al.*, 2017). Quantitative reverse-transcription PCR (qRT-PCR) analyses showed that *OsUGT90A1* was more highly expressed in leaves than in roots in both accessions, more highly expressed in leaves in the *japonica* than *indica* accession at warm and cold temperatures, and expression in both tissues increased in response to low temperature in both accessions (Fig. 2a). Similar results were obtained when *OsUGT90A1* expression data were averaged across three other chilling-tolerant RMC accessions containing the ACCTC haplotype (H57-3-1, WIR 911, and M202) and compared to three other chilling-sensitive accessions containing the GCTGG haplotype (Djimoron, IR 238, and Sapundali Local; Supplementary Fig. S2A). Thus, *OsUGT90A1* was more highly expressed in chilling-tolerant *japonica* than in chilling-sensitive *indica* accessions in all the RMC lines that we tested.

Because the first SNP (either A or G) of the five-SNP haplotype sequence of *OsUGT90A1* is within its proximate upstream regulatory region and overlaps with a potential bZIP binding site (ACTCAT) in *japonica* accessions (Supplementary Fig. S1) but is lacking a binding site in *indica* accessions due to a G at the same position (GCTCAT), in order to test expression differences we generated firefly luciferase (fLUC) reporter gene fusions using 500 bp of genomic DNA upstream of the *OsUGT90A1* coding region from Krasnodarskij 3352 (containing ACTCAT) and Carolino 164 (containing GCTCAT). Transgenic Arabidopsis plants with the ACTCAT-containing fragment (*JAP::LUC*) had significantly more fLUC expression after cold treatment than plants with the GCTCAT-containing fragment (*IND::LUC*; Fig. 2b, Supplementary Fig. S2B). Transient expression assays at room temperature in rice protoplasts showed that the ACTCA-containing fragment conferred higher fLUC expression than the GCTCA-containing fragment (Fig. 2c). Time-series analysis during cold treatment showed that expression of *OsUGT90A1* in both leaves and roots was initially up-regulated (0–7 h) and then down-regulated (Fig. 2d). Expression then increased again in roots after 48 h of cold treatment. Taken together, these results indicated that *OsUGT90A1* was more highly expressed in chilling-tolerant rice accessions than chilling-sensitive ones, and that SNPs in its upstream regulatory region might have been responsible for those differences in expression.

### Involvement of *OsUGT90A1* in protecting cell membrane integrity during abiotic stress

**Fig. 3.**
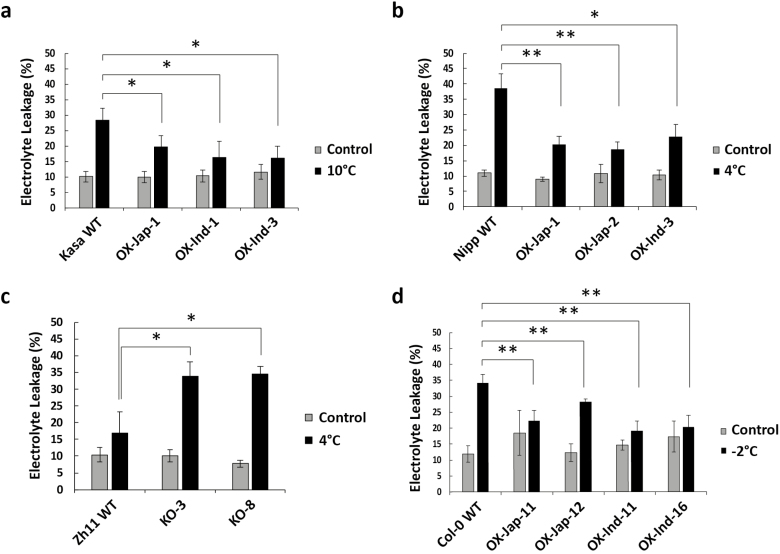
Determination of electrolyte leakage (EL) in leaves as a measure of cellular damage induced by low-temperature stress in wild-type (WT) and *OsUGT90A1*-overexpression (OX) and knockout (KO) transgenic rice and Arabidopsis plants. (a) EL in the WT *indica* accession Kasalath (Kasa) and in three *OsUGT90A1*-OX lines grown under control conditions (28/25 °C day/night, 12-h photoperiod) or after chilling treatment at 10 °C for 2 d. (b) EL in the WT *japonica* accession Nipponbare (Nipp) and in three *OsUGT90A1*-OX transgenic lines grown under control conditions or after chilling treatment at 4 °C for 4 d. (c) EL in the WT *japonica* accession Zhong Hua 11 (ZH11) and in two *OsUGT90A1*-KO lines grown under control conditions or after chilling treatment at 4 °C for 4 d. (d) EL in the Arabidopsis WT Col-0 and in four transgenic lines overexpressing *OsUGT90A1* (OX) grown under control conditions (22/20 °C, 16/8 h day/night) or after freezing treatment at –2 °C for 1.5 h. Ind- and Jap- indicate that the *indica* or *japonica* allele of *OsUGT90A1* was overexpressed, respectively. Data are means (±SD) of *n*=3 assays. Significant differences between means are indicated and were determined using two-tailed Student’s *t*-tests: *P<0.05; **P<0.01; ***P<0.001.

**Fig. 4.**
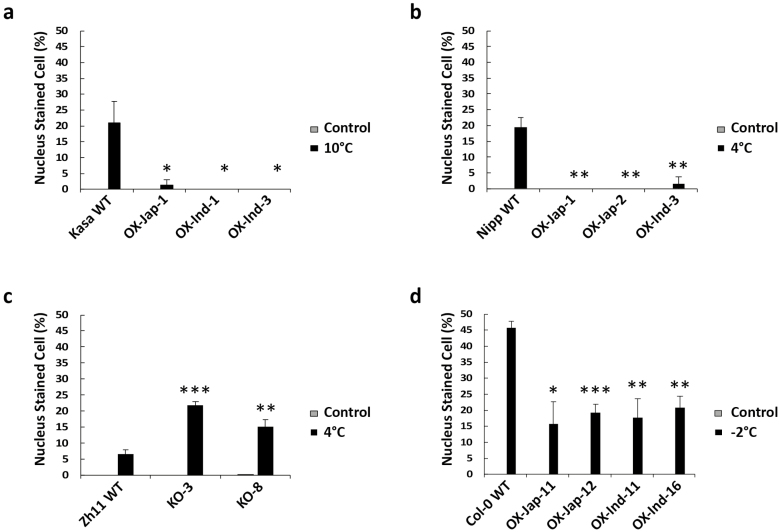
Determination of propidium iodide (PI) staining of nuclei in epidermal tissues of rice and Arabidopsis leaves using laser confocal microscopy. Nuclear staining indicates plasma membrane damage. (a) Percentage of cells with stained nuclei in the wild-type (WT) *indica* accession Kasalath (Kasa) and in three *OsUGT90A1*-overexpression (OX) transgenic lines grown under control conditions (28/25 °C day/night, 12-h photoperiod) or after chilling treatment at 10 °C for 2 d. (b) Percentage of cells with stained nuclei in the WT *japonica* accession Nipponbare (Nipp) and in three *OsUGT90A1*-OX transgenic lines grown under control conditions or after chilling treatment at 4 °C for 4 d. (c) Percentage of cells with stained nuclei in the WT *japonica* accession Zhong Hua 11 (ZH11) and in two *OsUGT90A1*-knockout (KO) lines grown under control conditions or after chilling treatment at 4 °C for 4 d. (d) Percentage of cells with stained nuclei in WT Arabidopsis Col-0 and in four transgenic lines overexpressing *OsUGT90A1* (OX) grown under control conditions (22/20 °C, 16/8 h day/night) or after freezing treatment at –2 °C for 1.5 h. Ind- and Jap- indicate that the *indica* or *japonica* allele of *OsUGT90A1* was overexpressed, respectively. Significant differences between the WT and the transgenic lines were determined using two-tailed Student’s *t*-tests: *P<0.05; **P<0.01; ***P<0.001. Note that almost no staining was observed under control conditions.

Increased *OsUGT90A1* expression might contribute to the superior chilling tolerance of *japonica* accessions; however, it might also be due to differences in the activity of the OsUGT90A1 enzyme resulting from differences in the protein sequences (Supplementary Fig. S1C). To test these possibilities, we made transgenic rice plants overexpressing either the *japonica* allele of *OsUGT90A1* (from Krasnodarskij 3352) or the *indica* allele (from Carolino 164) from a strong constitutive promoter in both the chilling-tolerant Nipponbare and chilling-sensitive Kasalath backgrounds, and knocked the gene out in the chilling-tolerant Zhong Hua 11 (ZH11) background. We also overexpressed either allele in transgenic Arabidopsis lines to assess their effects in a heterologous system. An analysis of three independent overexpression (OX) transgenic lines with constitutively high *OsUGT90A1* mRNA levels for each background and two knockout (KO) lines with very low mRNA levels (Supplementary Fig. S3) showed that there were no significant differences in the LTSS scores between the wild-type (WT) controls and transgenic lines under cold-stress conditions (Supplementary Fig. S2C–E). However, overexpression of either *OsUGT90A1* allele in transgenic Arabidopsis lines improved their freezing survival, and the temperature at which 50% of seedlings died shifted from –3 °C to –4 °C (Supplementary Fig. S2F). In contrast, when we determined percent electrolyte leakage (%EL) as a measure for cellular membrane damage induced by cold stress (e.g. Demidchik *et al.*, 2014), there were significant differences between the WT and transgenic lines (Fig. 3a–c). Regardless of which allele was used, after exposure to cold the overexpression lines had lower %EL values than the WT and the knockout lines had higher values, suggesting that *OsUGT90A1* helped to protect plasma membranes against cellular lesions induced by cold stress. The same membrane protection effect was observed in *OsUGT90A1*-OX Arabidopsis lines exposed to a mild freezing temperature (Fig. 3d). Because both the *japonica* and *indica* coding sequences of *OsUGT90A1* had similar protecting effects, it is more likely that differences in expression rather than differences in enzyme activity were responsible for the observed differences in chilling-tolerance phenotypes. Consistent with this, there was a general trend for a negative correlation between *OsUGT90A1* expression levels and %EL in transgenic rice and Arabidopsis plants (Supplementary Fig. S4). To validate the EL results, we stained WT, OX, and KO lines with propidium iodide, which cannot cross an intact plasma membrane and generates a fluorescent signal in the nucleus only after penetrating damaged plasma membranes (Chen *et al.*, 2006). The results confirmed that after exposure to cold the *OsUGT90A1*-OX lines had fewer plasma membrane lesions compared to the WT and *OsUGT90A1*-KO lines had more (Fig. 4, Supplementary Fig. S5).

### Involvement of *OsUGT90A1* in abiotic stress tolerance pathways

**Fig. 5.**
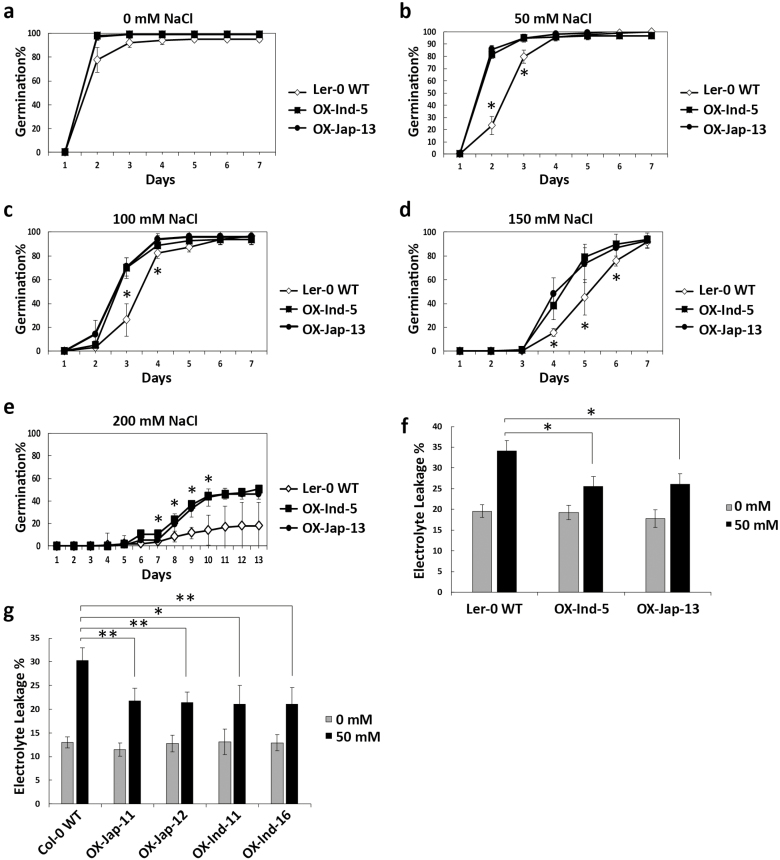
Determination of tolerance to salt stress in Arabidopsis wild-type (WT) ecotypes and in transgenic lines overexpressing *OsUGT90A1* (OX). (a–e) Time-course of seed germination of WT and two *OsUGT90A1*-OX homozygous transgenic lines on half-strength MS medium supplemented with 0, 50, 100, 150, or 200 mM NaCl. (f, g) Electrolyte leakage in 2-week-old (f) Landsberg *erecta*-0 (L*er*-0) and (g) Columbia-0 (Col-0) Arabidopsis ecotypes in WT and *OsUGT90A1*-OX transgenic lines grown with 0 mM or 50 mM NaCl. Ind- and Jap- indicate that the *indica* or *japonica* allele of *OsUGT90A1* was overexpressed, respectively. Data are means (±SD) of *n*=3 assays. Significant differences between the WT and transgenic plants were determined using two-tailed Student’s *t*-tests: *P<0.05; **P<0.01.

Multiple abiotic stress response mechanisms often converge in a network of coordinated biochemical pathways. Because *OsUGT90A1* has previously been shown to be up-regulated by salt stress and to fall within the boundaries of a mapped salt-tolerance QTL (Saravana Kumar *et al.*, 2014), we tested *OsUGT90A1*-OX Arabidopsis lines in response to salt stress using an established germination assay (Xu *et al.*, 2011). This showed that while germination rates were similar on 0 mM NaCl control plates (Fig. 5a), there were several time-points during a 2-week germination period with significant differences on 50–200 mM NaCl for which seeds of transgenic lines germinated faster than those of the WT, regardless of which *OsUGT90A1* allele was overexpressed (Fig. 5b–e). Moreover, *OsUGT90A1*-OX lines also experienced less membrane damage on 50 mM NaCl than WT plants (Fig. 5f, g), indicating that *OsUGT90A1* also affected the mechanisms of tolerance to salt stress.

**Fig. 6.**
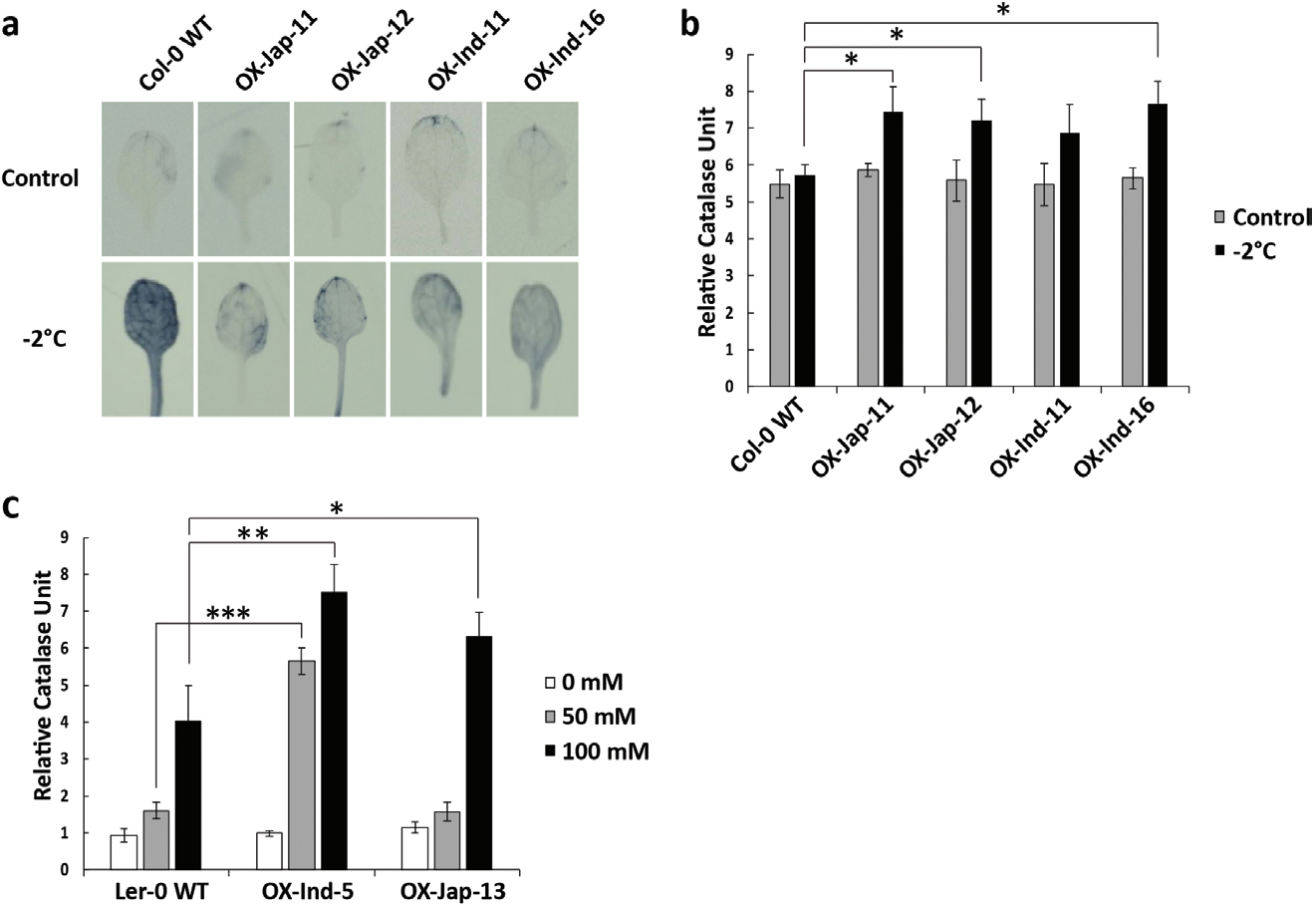
Visualization of reactive oxygen species (ROS) and activity of the antioxidant enzyme catalase (CAT) in Arabidopsis wild-type (WT) and transgenic lines overexpressing *OsUGT90A1* (OX). (a) Staining with Nitro Tetrazolium Blue to visualize O_2_^•–^ in WT Columbia-0 (Col-0) and in four *OsUGT90A1*-OX transgenic lines under control temperatures (22/20 °C, 16/8 h day/night) or after freezing stress at –2 °C for 1.5 h. (b) CAT activity in WT Col-0 plants and in four *OsUGT90A1*-OX transgenic lines under control conditions or after freezing stress at –2 °C for 1.5 h. (c) CAT activity in WT Landsberg *erecta*-0 (L*er*-0) plants and in two *OsUGT90A1*-OX transgenic lines grown with 0, 50, or 100 mM NaCl. Ind- and Jap- indicate that the *indica* or *japonica* allele of *OsUGT90A1* was overexpressed, respectively. Significant differences between the WT and transgenic plants were determined using two-tailed Student’s *t*-tests: *P<0.05; **P<0.01; ***P<0.001. (This figure is available in colour at *JXB* online.)

Because ROS are produced during abiotic stress, we determined whether *OsUGT90A1* affected their levels during cold stress. Staining with NBT, which qualitatively detects ROS in living cells (Muñoz *et al.*, 2000; Kumar *et al.*, 2015), showed that while there were no differences between the WT and *OsUGT90A1*-OX or *Osugt90a1*-KO transgenic rice plants at warm temperatures, at cold temperatures both *OsUGT90A1*-OX rice and Arabidopsis plants had fewer ROS-producing regions in the leaves than WT plants while *Osugt90a1*-KO rice plants had more and larger regions (Fig. 6a, Supplementary Fig. S6).

**Fig. 7.**
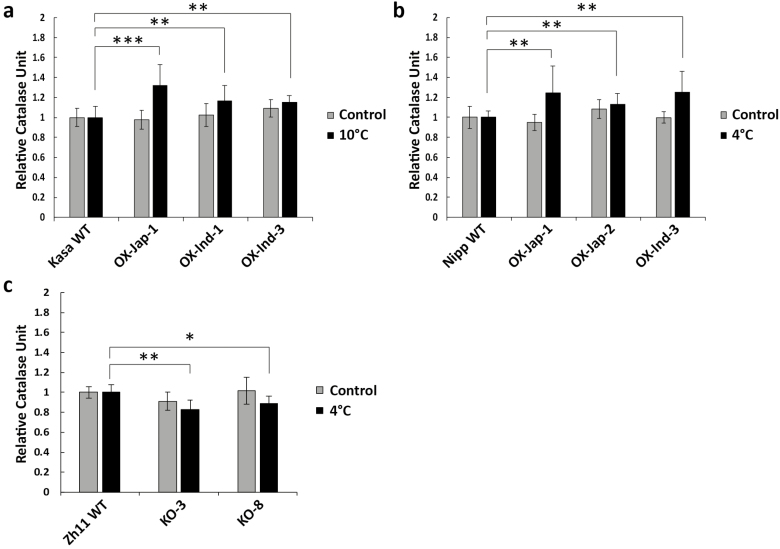
Activity of the antioxidant enzyme catalase (CAT) in rice wild-type (WT) and transgenic *OsUGT90A1*-overexpression (OX) lines. (a) CAT activity in WT Kasalath (Kasa) and three *OsUGT90A1*-OX lines at warm temperatures (control; 28/25 °C day/night, 12-h photoperiod) or after chilling stress at 10 °C for 2 d. (b) CAT activity in WT Nipponbare (Nipp) and three *OsUGT90A1*-OX lines under control conditions or after chilling stress at 4 °C for 4 d. (c) CAT activity in WT Zhong Hua 11 (ZH11) and two *Osugt90a1*-knockout (KO) lines under control conditions or after chilling treatment at 4 °C for 4 d. Ind- and Jap- indicate that the *indica* or *japonica* allele of *OsUGT90A1* was overexpressed, respectively. Data are means (±SD) of *n*=3 assays and the values are expressed relative to those of the WT controls, which were set as 1. Significant differences between the WT and transgenic plants were determined using two-tailed Student’s *t*-tests: *P<0.05; **P<0.01; ***P<0.001.

**Fig. 8.**
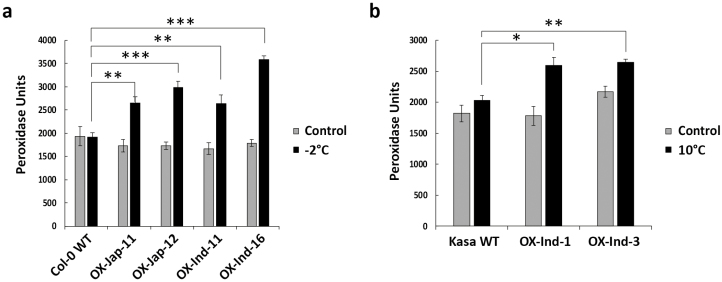
Activity of the antioxidant enzyme peroxidase (POX) in wild-type (WT) and transgenic *OsUGT90A1*-overexpression (OX) Arabidopsis and rice lines. (a) POX activity in Arabidopsis WT Col-0 and in four *OsUGT90A1*-OX lines at warm temperatures (control; 28/25 °C day/night, 12-h photoperiod) or after freezing stress at –2 °C for 1.5 h. (b) POX activity in rice WT Kasalath (Kasa) and in two *OsUGT90A1*-OX lines under control conditions or after chilling stress at 10 °C for two days. Ind- and Jap- indicate that the *indica* or *japonica* allele of *OsUGT90A1* was overexpressed, respectively. Data are means (±SD) of *n*=3 assays. Significant differences between the WT and transgenic plants were determined using two-tailed Student’s *t*-tests: *P<0.05; **P<0.01; ***P<0.001.

To determine whether overexpression of *OsUGT90A1* affected enzymatic antioxidant activity during cold stress, we examined the activities of catalase (CAT) and peroxidase (POX) as a measure for H_2_O_2_ scavenging (Willekens *et al.*, 1997; Mittler *et al.*, 2004). Both assays showed that there were no differences between the WT and transgenic plants at warm temperatures (Figs 6b, 7, 8); however, under cold-stress conditions that resulted in more ROS (Fig. 6a, Supplementary Fig. S6), there was higher CAT and POX activity in both Arabidopsis and rice *OsUGT90A1*-OX lines (Figs 6b, 7a, b, 8), and lower CAT activity in *Osugt90a1*-KO lines (Fig. 7c). Moreover, while there were no differences in CAT activity between the WT and *OsUGT90A1*-OX Arabidopsis plants under control conditions, activity was generally higher in *OsUGT90A1*-OX plants grown for 2 weeks on 50 mM and 100 mM NaCl (Fig. 6c). The higher CAT and POX activities correlated well with a reduction in the leaf-bleaching (necrosis) phenotype that is observed in rice seedlings damaged by ROS during recovery growth after stress treatment (Chen *et al.*, 2012), with *OsUGT90A1*-OX plants having smaller areas of necrosis than WT control plants (Supplementary Fig. S7). Hence, overexpression of *OsUGT90A1* during cold stress correlated with lower levels of ROS and increased antioxidant enzyme activities, resulting in decreased membrane lesions in response to abiotic stress.

### Subcellular localization OsUGT90A1 and assessment of its potential function

**Fig. 9.**
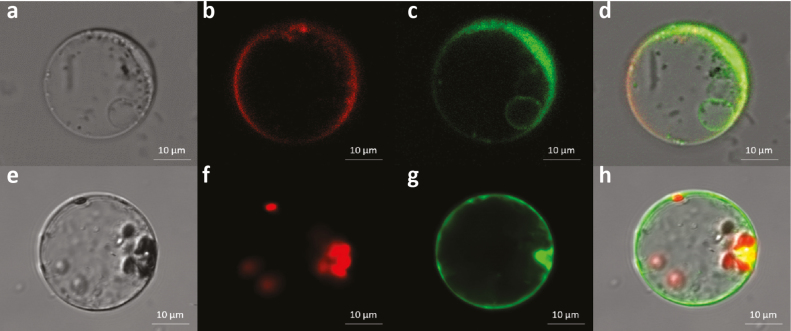
Visualization of the subcellular localization of OsUGT90A1::eGFP (enhanced green fluorescent protein) in rice protoplasts at warm temperatures (28/25 °C day/night, 12-h photoperiod). (a) Bright-field image of a protoplast containing the OsUGT90A1::eGFP fusion protein. (b) Staining with FM^TM^ 4–64 dye to visualize the plasma membrane (red). (c) Visualization of the OsUGT90A1::eGFP fusion protein (green). (d) Merged image of (b) and (c) to visualize areas of overlap (yellow). The small green ring inside the protoplast shows an affinity of OsUGT90A1::eGFP for an internal membrane, most likely the tonoplast. (e) Bright-field image of a protoplast containing the OsUGT90A1::eGFP fusion protein. (f) Visualization of chloroplasts (red). (g) Visualization of the OsUGT90A1::eGFP fusion protein (green). (h) Merged image of (f) and (g) to visualize areas of overlap (yellow).

The subcellular localization of a protein can help in inferring its function. To determine the localization of OsUGT90A1, we introduced plasmids containing N-terminal fusions of the gene to the eGFP reporter into rice protoplasts. Examination of numerous eGFP-positive protoplasts using confocal laser-scanning microscopy showed that the fusion protein predominantly localized to the cytoplasm, but also had some affinity for the plasma membrane and internal membrane systems such as the tonoplast (Fig. 9c, d) but not the nucleus or chloroplasts (Fig. 9g, h). This suggested that OsUGT90A1 performs its function predominantly in the cytoplasm and/or in association with internal membranes.

To determine whether differential expression of *OsUGT90A1* (which is annotated as a putative anthocyanin 3-O-beta-glucosyltransferase) affected the overall anthocyanin concentration, we measured the total flavonoid/anthocyanin contents in leaf tissues of plants grown with or without chilling stress, and the results indicated that there were no differences between the WT and transgenic plants (Supplementary Fig. S8A, B). To test whether *OsUGT90A1* affected the abundance of particular flavonoid/anthocyanin compounds without changing the total content, we performed HPLC-MS analyses to examine their specific compositions in the leaf tissues of the WT, and OX and KO transgenic lines immediately after chilling treatment. Compared to WT plants, there was a slightly different flavonoid/anthocyanin composition profile in *OsUGT90A1*-OX transgenic plants (Supplementary Fig. S9), but no clear differences were detected between the WT and *Osugt90a1*-KO plants (Supplementary Fig. S10). To determine whether the slightly different profiles in the OX lines had an effect on the non-enzymatic antioxidant activity of the total flavonoid/anthocyanin compounds that we isolated, we performed a DPPH reduction assay. DPPH can be reduced by common antioxidants and therefore acts as an indicator for general antioxidant activity (Molyneux, 2004). We found that there were no differences in the antioxidant activity between the WT and the *OsUGT90A1*-OX lines (Supplementary Fig. S8C, D), suggesting that the observed increases in ROS-scavenging activity of the OX plants were not due to the slightly altered flavonoid/anthocyanin composition profiles.

**Fig. 10.**
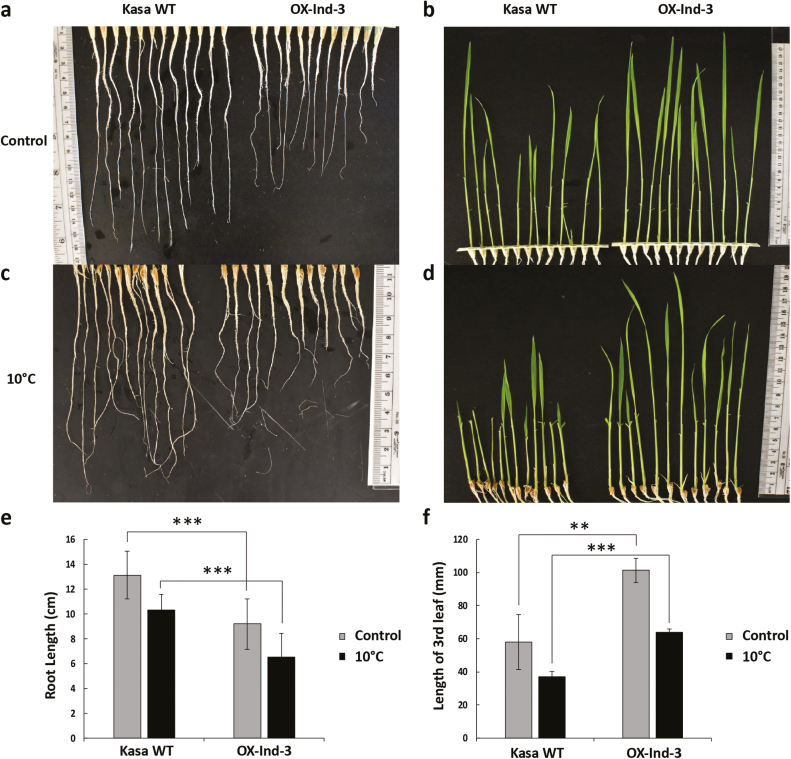
Comparison of rice wild-type (WT) and a transgenic line overexpressing the *indica* allele of *OsUGT90A1* (OX-Ind-3), showing a phenotype of short roots and long shoots in the latter. (a, c) Roots of 22-d-old WT Kasalath (Kasa) and *OsUGT90A1*-OX plants grown under (a) warm temperature conditions (control; 28/25 °C day/night, 12-h photoperiod), or (c) after chilling stress at 10 °C for 2 d. (b, d) The third leaf of 22-d-old Kasa WT and *OsUGT90A1*-OX plants grown under warm temperature growth conditions (control) or (d) after chilling stress at 10 °C for 2 d. The first and second leaves were removed for clarity. (e) Quantification of root lengths shown in (a) and (c). (f) Quantification of leaf lengths shown in (b) and (d). Ind- indicates that *OsUGT90A1* was overexpressed. Data are means (±SD) of *n*=12 plants. Significant differences between the WT and transgenic plants were determined using two-tailed Student’s *t*-tests: **P<0.01; ***P<0.001.

We looked next for growth and development phenotypes as indicators for other potential enzyme substrates. UGTs have previously been shown to regulate phytohormone homeostasis by glycosylation (Jackson *et al.*, 2001; Xu *et al.*, 2002; Hou *et al.*, 2004). Since phytohormones are critical plant growth regulators, we determined whether root and shoot lengths were affected by differential expression of *OsUGT90A1*. Interestingly, *OsUGT90A1*-OX rice plants had longer shoots but shorter roots than control WT plants, both under warm-temperature control conditions and under cold stress (Fig. 10). This phenotype suggested that the presumed glycosylation activity of OsUGT90A1 might have a direct or indirect effect on phytohormones such as auxins and cytokinins.

## Discussion

To the best of our knowledge, we present the first evidence that a gene annotated to encode a UDP-glycosyltransferase, *LOC_Os04g24110* (=*OsUGT90A1*), plays a role in chilling tolerance of rice. *OsUGT90A1* is one of the probable candidate genes associated with the chilling-tolerance QTL *qLTSS4-1*(Schläppi *et al.*, 2017), and our functional assays revealed a role in protecting the plasma membrane from lesions induced by cold stress. Accessions from both the *indica* and *japonica* subspecies in which *OsUGT90A1* was overexpressed experienced less membrane damage after cold stress than wild-type (WT) plants, while a generally cold-tolerant accession in which the gene was knocked out experienced more membrane damage (Fig. 3). However, although Arabidopsis lines overexpressing *OsUGT90A1* had better freezing survival abilities than WT plants, transgenic rice lines did not have improved low-temperature seedling survivability (LTSS) scores; this might be, at least in part, because rice chilling tolerance is a highly quantitative trait. We have previously shown that each QTL contributes only 2–20% to any of the studied chilling-tolerance indices, indicating that these are polygenic traits (Schläppi *et al.*, 2017). This is also in agreement with the recently proposed omnigenic theory, which suggests that almost any gene can influence a complex trait (Boyle *et al.*, 2017). It is therefore remarkable that in the current study overexpression or knockout of a single enzyme-encoding gene had such a measurable effect on plasma membrane integrity, which is essential for plant survival during exposure to abiotic stress (Kim and Tai, 2011). Thus, *OsUGT90A1* has a significant auxiliary role and could be used to improve chilling tolerance of rice without affecting major gene regulons, which is a major concern when transcription factors or signaling molecules are used to improve the performance of plants under abiotic stress. Taken together, our results indicate that *OsUGT90A1* is probably a major candidate gene associated with *qLTSS4-1*; however, due to the polygenic nature of chilling tolerance, we cannot rule out that other genes within the *qLTSS4-1* boundary contribute to this QTL.

*OsUGT90A1* was more highly expressed in *japonica* than *indica* accessions (Fig. 2). A reason for this differential expression might be the A/G SNP at –289 from the start of the coding region. At this position, almost all *japonica* accessions tested had an A, which generates a potential ACTCAT *cis*-element for binding of Group-S basic leucine zipper (bZIP) factors (Satoh *et al.*, 2002), while the majority of *indica* accessions had a G. It has previously been shown that the Group-S factor bZIP73 plays a major role in the superior chilling tolerance of *japonica* accessions, because a single amino acid substitution reduces the bZIP73 *trans*-activation potential in *indica* accessions (Liu *et al.*, 2018). The lower abundance of *OsUGT90A1* mRNA in *indica* than *japonica* accessions that we consistently observed might be in part due to the absence of a bZIP73-interacting ACTCAT element and/or the presence of a bZIP73 protein with reduced *trans*-activation potential. Consistent with this, in our transient expression assays the ACTCAT-containing promoter element produced only 1.2-fold higher reporter gene expression than the GCTCAT one, which may have been because the assays were done in protoplasts derived from the 93-11 accession, which contains a bZIP73 protein with reduced *trans*-activation potential (Liu *et al.*, 2018).

The positive effect of *OsUGT90A1* in reducing membrane lesions induced by abiotic stress was validated in the heterologous system of Arabidopsis (Fig. 3d). Thus, *OsUGT90A1* had a similar auxiliary role in both a monocot and a dicot species. Arabidopsis salt germination assays suggested that the *OsUGT90A1*-overexpressing (-OX) lines had lower levels of abscisic acid (ABA), because they germinated faster than WT plants on salt (Fig. 5) whereas salt stress generally increases ABA levels to repress germination (Xu *et al.*, 2011). In contrast, activities of the antioxidant enzyme catalase (CAT) were higher in the OX than WT plants when exposed to salt and during freezing stress, but levels of reactive oxygen species (ROS) were lower in the OX plants than in the WT (Fig. 6). Similarly, ROS levels were lower and CAT and peroxidase (POX) levels were higher in OX plants than in the WT during chilling stress in rice (Figs 7, 8).

How overexpression of *OsUGT90A1* enhances antioxidant enzyme activities to reduce ROS levels during abiotic stress is currently unknown. It is tempting to speculate that OsUGT90A1 glycosylates metabolites that are involved in balancing abiotic stress responses with the general requirement for growth and development. Although *OsUGT90A1* is annotated to encode an anthocyanin 3-O-beta-glucosyltransferase, we detected no significant alterations in either the total content or composition of flavonoid/anthocyanin metabolites (Supplementary Figs S8–S10), indicating that flavonoids and/or anthocyanins might not be the actual substrates for the OsUGT90A1 enzyme.

An alternative hypothesis is that OsUGT90A1 glycosylates phytohormones, which generally leads to the sequestration of hormone glycosides into storage organelles (Mok and Mok, 2001). Chilling-tolerant rice accessions have been shown to have lower ABA levels than chilling-sensitive ones, which balances stress responses with growth and development (Li *et al.*, 2018). Hence, OsUGT90A1 might help to control the amount of active plant hormones by forming sugar conjugates and might help to regulate redox status through hormone-mediated pathways, which may allow harmless levels of ROS to function as signaling molecules (Xia *et al.*, 2015; Tognetti *et al.*, 2017). The crosstalk between plant hormones (e.g. auxin and cytokinin) and ROS plays an important role in regulating plant growth and development. When plants are exposed to environmental stresses, RESPIRATORY BURST OXIDASE HOMOLOG (RBOH) is activated to produce signaling ROS, such as H_2_O_2_, which work together with mitogen-activated protein kinases (MAPKs) and Ca^2+^-dependent protein kinases (CDPKs) to regulate stress tolerance responses (e.g. Abbasi *et al.*, 2004). Phytohormones such as auxins and ABA generate positive feedback for this signaling pathway and produce more ROS via an auxin- or ABA-receptor mediated activation of the RBOH mechanism (Xia *et al.*, 2015). This auxin/ABA–ROS positive feedback loop might be modulated by OsUGT90A1-mediated glycosylation of active auxin and/or ABA molecules.

We further showed that overexpression of *OsUGT90A1* in rice inhibited root development and enhanced shoot development (Fig. 10). This further suggests that *OsUGT90A1* might modulate the activity of general growth regulators. Although the effect of overexpression on root and shoot growth was different, it was consistent with the known antagonistic interactions of auxins and cytokinins, such that a low ratio of auxin:cytokinin leads to shorter roots but longer shoots (Dello Ioio *et al.*, 2008; Schaller *et al.*, 2015). Likewise, ABA and gibberellic acid (GA) have antagonistic effects on germination (Vishal and Kumar, 2018). Overexpression of *OsUGT90A1* in Arabidopsis increased seed germination rates under salt-stress conditions (Fig. 5). This was consistent with the hypothesis that auxins and/or ABA rather than anthocyanins might be substrates for OsUGT90A1. In this model, reduced activities of auxins and/or ABA due to glycosylation-mediated sequestration into vacuoles would result in shorter roots, longer leaves, and improved germination rates.

Interestingly, although there were amino acid substitutions between *japonica* and *indica* haplotypes, overexpression of either haplotype had a similar positive effect on reducing cellular lesions induced by abiotic stress (Figs 3–5). Thus, naturally occurring ‘overexpression’ of *OsUGT90A1* in *japonica* accessions under both warm and cold stress conditions (Fig. 2) most likely results in high OsUGT90A1 protein levels and plays a positive role in balancing abiotic stress responses with the requirement of low ROS levels and an intact cellular membrane in preparation for growth and development. However, we cannot rule out that amino acid substitutions have a subtle effect on the function of OsUGT90A1. On the other hand, *OsUGT90A1* was recently assigned to the ‘down-regulated’ core transcriptome response induced by abiotic stress in rice (Cohen and Leach, 2019), most likely because after the initial up-regulation during cold stress, *OsUGT90A1* may be down-regulated to fine-tune its effect on growth and development.

## Conclusions

We propose the following model for the auxiliary role of *OsUGT90A1* in the chilling-stress response of rice. Cold-tolerant *japonica* accessions have higher mRNA abundance of *OsUGT90A1* than cold-sensitive *indica* accessions, and transcript abundance increases (at least temporarily) during exposure to cold temperatures, most likely leading to an increase in abundance of the OsUGT90A1 protein. OsUGT90A1 might glycosylate ABA and/or auxins to sequester them into the vacuole for storage. The consequence of this is a fine-tuning of the ABA- and/or auxin-mediated abiotic stress response, leading to an increase in the activities of CAT, POX, and possibly other antioxidant enzymes. This in turn reduces ROS levels and thus maintains cell membrane integrity as a general mechanism of abiotic stress tolerance to help the resumption of shoot growth and development during subsequent stress recovery.

## Supplementary data

Supplementary data are available at *JXB* online.

Fig. S1. SNP haplotypes of the *LOC_Os04g24110* promoter, coding region, and protein sequences in *japonica* and *indica* reference subspecies.

Fig. S2. *In vivo OsUGT90A1* promoter activity in response to cold stress, and the effect of *OsUGT90A1* expression on low-temperature seedling survivability in rice.

Fig. S3. Abundance of *UGT90A1* mRNA in the individual transgenic rice and Arabidopsis plants used in Figs 3–8, 10, and Supplementary Fig. S4.

Fig. S4. Correlations of *OsUGT90A1* mRNA abundance with plasma membrane integrity in cold-stressed transgenic rice and Arabidopsis plants.

Fig. S5. Propidium iodide staining of wild-type and *OsUGT90A1*-OX and -KO transgenic rice and Arabidopsis lines.

Fig. S6. Effect of *OsUGT90A1* expression on ROS-scavenging activity under control and chilling-stress conditions in transgenic rice.

Fig. S7. Leaf-bleaching phenotype of wild-type rice and *OsUGT90A1*-overexpression plants.

Fig. S8. Overall concentrations and non-enzymatic antioxidant activities of total flavonoids/anthocyanins in leaves of wild-type rice and *OsUGT90A1*-overexpression plants.

Fig. S9. Effects of *OsUGT90A1* overexpression on flavonoid/anthocyanin composition under chilling stress in rice.

Fig. S10. Effects of *OsUGT90A1* knockout on flavonoid/anthocyanin composition under chilling stress in rice.

Table S1. List of primers used in this study.

eraa025_suppl_Supplementary_Figures_S1_S10_Table_S1
