# Supplementary material for: Glycosyltransferase OsUGT90A1 helps protect the plasma membrane during chilling stress in rice
Source: J Exp Bot. 2020 Jan 24;71(9):2723–39. doi: 10.1093/jxb/eraa025 (PMC7210772; doi:10.1093/jxb/eraa025)
Supplement: eraa025_suppl_Supplementary_Figures_S1_S10_Table_S1 [file eraa025_suppl_supplementary_figures_s1_s10_table_s1.pdf]

Glycosyltransferase *OsUGT90A1* helps protect the plasma membrane during chilling stress in rice

Yao Shi,[a,d] Huy Phan[a], Yaju Liu,[b] Shouyun Cao,[c] Zhihua Zhang,[c] Chengcai Chu,[c] Michael R. Schläppi,[a*]

[a]Department of Biological Sciences, Marquette University, 1428 W Clybourn St, Milwaukee, WI 53233, USA. [b]National Sweet Potato Improvement Center, Sweet Potato Research Institute, Xuhai Rd., Xuzhou, 221131, P.R. China. [c]Institute of Genetics and Developmental Biology, Chinese Academy of Sciences, No.1 West Beichen Road, Beijing, 100101, P.R. China. [d]Current address: The University of Pennsylvania School of Dental Medicine, Levy building, Biochemistry Department, Rm538, 240 S 40th St, Philadelphia, PA 19104.

[*]**Correspondence: michael.schlappi@marquette.edu** +1 (414) 288-1480

Author contributions

MRS and YS conceived the project and designed the experiments. YS, HP, YL, ZZ, and MRS performed the experiments, and SC generated transgenic rice plants. CC provided material and intellectual support. YS and MRS performed all data analyses and wrote the manuscript. All authors edited and approved the final version of the manuscript.

a

```
Jap.  AGTAATTTACAATATAAATGTTTTTTCATCATAATATTATCATGTAAGATCTTGTTATAAAGATTTAATTGCAACGGACACAATGGTGTAATCGGATTAT   4000
Ind.  AGTAATTTACAATATAAATGTTTTTTCATCATAATATTATCATGTAAGATCTTGTTATAAAGATTTAATTGCAACGGACACAATGGTGTAATCGGATTAT   4000

Jap.  ATATCGGATAAGTAATTTAAGAGAAAATTTTATAAGAAGAAAAAAAATATGCACAACCTAATAGCAAAAACTACTTGCATGCATGTCTGTAACGCTACTA   4100
Ind.  ATATCGGATAAGTAATTTAAGAGAAAATTTTATAAGAAGAAAAAAAATATGCACAACCTAATAGCAAAAACTACTTGCATGCATGTCTGTAACGCTACTA   4100

Jap.  GTACTTCTCACGTAATTGTCCGCGTCGCTCGTTAAGTCTAAATCGAGATGCCTCTCGAAATAGATTTTGCGTAAATTATTCCCTCCTCCGTTCTAAAATA   4200
Ind.  GTACTTCTCACGTAATTGTCCGCGTCGCTCGTTAAGTCTAAATCGAGATGCCTCTCGAAATAGATTTTGCGTAAATTATTCCCTCCTCCGTTCTAAAATA   4200

Jap.  TTTTAATACAGAAGTAGTATTTAGCGTGTTAGGTAAGCTGCTTAAAAAAATTATGTAGTTATAATTTATTTTGGTATGAGTTGTTTTATCACTTAAGGTA   4300
Ind.  TTTTAATACAGAAGTAGTATTTAGCGTGTTAGGTAAGCTGCTTAAAAAAATTATGTAGTTATAATTTATTTTGGTATGAGTTGTTTTATCACTCAAGGTA   4300

Jap.  TTTAAAATTTTTGAATAAGACGAATGATCAAACATAAACATGTGTTCAAAAGTCAATGGTGTCATCGATTAAAAACCAGAGAGAGTAGATTTCAGGACAG   4400
Ind.  TTTAAAATTTTTGAATAAGACGAATGATCAAACATAAACATGTGTTCAAAAGTCAATGGTGTCATTGATTAAAAACCAGAGAGAGTAGATTTCAGGACAG   4400
                                                                                                  1
Jap.  AGGGAGTATTAAATTAGACACGTGTTTGTTATCATTTTTTTTGAAACAGTTGACGGTAAGATTTCTACCGGATATATTAGAAGAAAAGAGTTTTTACCTA   4500
Ind.  AGGGAGTATTAAATTAGACACGTGTTTGTTATCATTTTTTTTGAAACAGTTGACGGTAAGATTTCTACCGGATATATTAGAAGAAAAGAGTTTTCACCTA   4500
                                                        7
Jap.  AAGGGGGAGAGAGTTCACAAGGCAACACCTCCAAGGAGGAAACAGCACCCGCATACATCGATGTCACCGATCCTGTAAGACCAGACAAGGATTTCACCCA   4600
Ind.  AAGGGGGAGAGAGTTCACAAGGCAACACCTCCAAGGAGGAAACAGCACCCGCATGCGTCGATGTCACCGATCCTATAAGACCAGGCAAGGATTTCACCCA   4600

Jap.  AGACTGTCGCCGATCCTGTAAGACCAGGCAAGAATTTCACCTAAGACTACCTTAAAACGTGTAGAACCTTCAAGAAACAAGAGCAAAAGTGTTGAGTGTT   4700
Ind.  AGACTGTCGCCGATCCTGTAAGACCAGGCAAGAATTTCACCTAAGACTACCTTAAAACGTGTAGAACCTTCAAGAAACAAGAGCAAAAGTGTTGAGTGTT   4700
                 2   6     3              4                                                             5
Jap.  ATCGTTGCCCAACTCATCGGCTGACCCGATGCAGCAGCTCCGACTCCGCCTTAACACGAAAGCTCAAGCACCGTAGAGAAAAGCACACCGCTCCAAACTC   4800
Ind.  ATCGTTGCCCAGCTCATCGGTTGACCCGATGCAGCAGCTCCGACTCCGCCTTAACACGAAAGCTCAAGCACCGTAGAGAAAAGCACACCGCTCCAAACTT   4800

Jap.  GGCACTAAGACAGATCCACACAGTCACAGGGCTCTTATGCCTCGCCAGTGCTTTGTTATCATTTTGTACCATGTCTTTTGGGCCTACGGTGTACATTACT   4900
Ind.  GGCACTAAGACAGATCCACACAGTCACAGGGCTCTTATGCCTCGCCAGTGCTTTGTTATCATTTTGTACCATGTCTTTTGGGCCTACGGTGTACATTACT   4900
                                                                                               8
Jap.  ACATGTGGAGCGATTAGCAGCAGCCATTTCGTAGTTAGAATAGAAGAACACACTCTTGAGGCTATCAGCAACCAGCTCCACTGCTAGCTCCAGGCCGGTC   5000
Ind.  ACATGTGGAGCGATTAGCAGCAGCCATTTCGTAGTTAGAATAGAAGAACACACTCTTGAGGCTATCAGCAACCAGCTCCACTGCTAGCTCCAGGCCGGTC   5000

Jap.  ATGGCG                                                                                                 5006
Ind.  ATGGCG
```

Fig. S1. SNP haplotypes of LOC_Os04g24110 promoter, coding region, and protein sequences in *JAPONICA* and *INDICA* reference subspecies. (a) The three SNPs in promoter region used for the haplotype analysis in Fig. 1b-c are labeled 2, 3, and 4. Arrows 7 and 8 indicate forward and reverse cloning primers for promoter amplification. Lines with number indicate potential transcription factor binding sites. The line labeled 6 shows a potential *ACTCAT* bZIP73 binding site. The line labeled 4 shows a potential *CCGAC* Low Temperature Response Element (LTRE) binding site.

b

```
Jap.  ATGGCGGCGGCTGGTCACGATGTGCAGCTCCCCCACGTCGCCATCTTCCCGTTCATGGCCAGGGGCCACACCGTCCCGATGACCCACCTCGCCTGCCTTC     100
Ind.  ATGGCGGCGGCTGGTCACGATGTGCAGCTCCCCCACGTCGCCATCTTCCCGTTCATGGCCAGGGGCCACACCGTCCCGATGACCCACCTCGCCTGCCTTC     100

Jap.  TCCGCCGCCGCGGCCTCGCCACCGTCACCTTCTTCAGCACCCCGGGCAACGCGCCGTTCGTCCGGGGACAGCTCGACGACGACGTGGCCGTCGTGGAGCT     200
Ind.  TCCGCCGCCGCGGCCTCGCCACCGTCACCTTCTTCAGCACCCCGGGCAACGCGCCGTTCGTCCGGGGACAGCTCGACGACGACGTGGCCGTCGTGGAGCT     200

Jap.  TCCCTTCCCGGACCATGTCGTCGCCCGCGGCGCCGCGGAGTGCGTCGAGGCCCTCGACTCCCTGTTCCCCCTTCCGGCGTTCGTCGAGGCGGTGTCGGCG     300
Ind.  TCCCTTCCCGGACCATGTCGTCGCCCGCGGCGCCGCGGAGTGCGTCGAGGCCCTCGACTCCCTGTTCCCCCTTCCGGCGTTCGTCGAGGCGGTGTCGGCG     300

Jap.  CTCCGGCCGGGGCTGGAGGTGTCGCTCGCCGCCGCTCGGCCCCGCGTCGGCCTCCTCGTCGCCGACGCGTTCCTGCACTGGGCGCACGCCTCCGCCGCCG     400
Ind.  CTCCGGCCGGGGCTGGAGGTGTCGCTCGCCGCCGCTCGGCCCCGCGTCGGCCTCCTCGTCGCCGACGCGTTCCTGCACTGGGCGCACGCCTCCGCCGCCG     400

Jap.  CGCTCGGCGTCCCGACGGTGGCGTTCCTCGGCGGGAACATGTTCGCGACGATCATGCGCGACGTGATCCTCCGCGACAACCCGGCCGCCGCGCTGCTGTC     500
Ind.  CGCTCGGCGTCCCGACGGTGGCGTTCCTCGGCGGGAACATGTTCGCGACGATCATGCGCGACGTGATCCTCCGCGACAACCCGGCCGCCGCGCTGCTGTC     500

Jap.  TGGCGGCGGCGGCGCCGAGGCGGCGACGTTCGCCGTGCCGGAGTTCCCGCACGTCCACCTCACGCTCGCCGACATCCCGGTCCCCTTCAACCGCCCGTCC     600
Ind.  TGGCGGCGGCGGCGCCGAGGCGGCGACGTTCGCCGTGCCGGAGTTCCCGCACGTCCACCTCACGCTCGCCGACATCCCGGTCCCCTTCAACCGCCCGTCC     600

Jap.  CCGGAGGGTCCGATCATGGAGCTGAACGCCAAGCTATGGAAGGCCATCGCCGGCAGCAACGGCCTCATCGTCAACACCTTCGACGCCATGGAAGGCCGCT     700
Ind.  CCGGAGGGTCCGATCATGGAGCTGAACGCCAAGCTATGGAAGGCCATCGCCGGCAGCAACGGCCTCATCGTCAACACCTTCGACGCCATGGAAGGCCGCT     700

Jap.  ACGTCGAGCACTGGAACCGTGACCACCGCGCCGGCCCCAGGGCGTGGCCCATAGGCCCGCTCTGCCTCGCCCATGGCGGCACCGGCACCGGCACCGGCGC     800
Ind.  ACGTCGAGCACTGGAACCGTGACCACCGCGCCGGCCCCAGGGCGTGGCCCATAGGCCCGCTCTGCCTCGCCCATGGCGGCACCGGCACCGGCACCGGCGC     800

Jap.  CGTCGAGCCCTCATGGATGAAGTGGCTGGACGAGAAGGCGGCCGCCGGGAGAGCCGTGCTGTACGTGGCGCTCGGCACCGCCATGGCCATCCCGGACGCG     900
Ind.  CGTCGAGCCCTCATGGATGAAGTGGCTGGACGAGAAGGCGGCCGCCGGGAGAGCCGTGCTGTACGTGGCGCTCGGCACCGCCATGGCCATCCCGGACGCG     900
                                                         1
Jap.  CAGCTCAGGGAGGTCGCCGGCGGGCTGGAAGCCGCCGCGGCCGCGGGCGTCTACTTCCTGTGGGCCGTGCGCCCCAGCGACGCCGACCTCGGCGCCGGCT    1000
Ind.  CAGCTCAGGGAGGTCGCCGGCGGGCTGGAAGCCGCCGCGGCCGCGGGCGTCGACTTCCTGTGGGCCGTGCGCCCCAGCGACGCCGACCTCGGCGCCGGCT    1000

Jap.  TCGAGGAGCGCGTGGAGGGGCGAGGCATGGTGGTGCGGGAGTGGGTGGACCAATGGCGGATCCTGCAGCACGGCTGCGTGAGGGGCTTCCTCAGCCACTG    1100
Ind.  TCGAGGAGCGCGTGGAGGGGCGAGGCATGGTGGTGCGGGAGTGGGTGGACCAATGGCGGATCCTGCAGCACGGCTGCGTGAGGGGCTTCCTCAGCCACTG    1100

Jap.  CGGGTGGAACTCGGCGGTGGAGGGCGTCGCCGCCGGCGTGCCGCTGGCGGCGTGGCCGATGGGCGCCGAGCAGCCGCTCAACGCGATGCTCGTCGTCGAC    1200
Ind.  CGGGTGGAACTCGGCGGTGGAGGGCGTCGCCGCCGGCGTGCCGCTGGCGGCGTGGCCGATGGGCGCCGAGCAGCCGCTCAACGCGATGCTCGTCGTCGAC    1200

Jap.  GAGCTGCGCGTCGGGGTCAGGGTGCCGGTGCCGACGGCGATGGCGACGGGCGGGCACGGGGTGGTCGGGAGCGAGGTGATCGCGCGCGTGGCGAGGGAGC    1300
Ind.  GAGCTGCGCGTCGGGGTCAGGGTGCCGGTGCCGACGGCGATGGCGACGGGCGGGCACGGGGTGGTCGGGAGCGAGGTGATCGCGCGCGTGGCGAGGGAGC    1300
                                                 2                3          4
Jap.  TGATGATGATGGCCGGGGAGGGGAAGGGCGGCGGCGGCGGCGAGGAGGCGAGGAACGTCGCCGCGCTGGCGTCCAAGGCGCGAGAAGCGGTCGCCGAGGG    1400
Ind.  TGATGATGATGGCCGGGGAGGGGAAGGGCGGCGGCGGCGGCGAGGCGGCGAGGAACGTCGCCTCGCTGGCGTCGAAGGCGCGAGAAGCGGTCGCCGAGGG    1400

Jap.  CGGGTCGTCGTGGAAGGCGCTGGAGGAGATGGTTGCCACGCTCTGCCGCCCTGTTGAAGGAGATACACCGAAACCCACAAAATAANN                  1487
Ind.  CGGGTCGTCGTGGAAGGCGCTGGAGGAGATGGTTGCCACGCTCTGCCGCCCTGTTGAAGGAGATACACCGAAACCCACAAAATAANN                  1487
```

c

```
Jap.  MAAAGHDVQLPHVAIFPFMARGHTVPMTHLACLLRRRGLATVTFFSTPGNAPFVRGQLDDDVAVVELPFPDHVVARGAAECVEALDSLFPLPAFVEAVSA     100
Ind.  MAAAGHDVQLPHVAIFPFMARGHTVPMTHLACLLRRRGLATVTFFSTPGNAPFVRGQLDDDVAVVELPFPDHVVARGAAECVEALDSLFPLPAFVEAVSA     100

Jap.  LRPGLEVSLAAARPRVGLLVADAFLHWAHASAAALGVPTVAFLGGNMFATIMRDVILRDNPAAALLSGGGGAEAATFAVPEFPHVHLTLADIPVPFNRPS     200
Ind.  LRPGLEVSLAAARPRVGLLVADAFLHWAHASAAALGVPTVAFLGGNMFATIMRDVILRDNPAAALLSGGGGAEAATFAVPEFPHVHLTLADIPVPFNRPS     200

Jap.  PEGPIMELNAKLWKAIAGSNGLIVNTFDAMEGRYVEHWNRDHRAGPRAWPIGPLCLAHGGTGTGTGAVEPSWMKWLDEKAAAGRAVLYVALGTAMAIPDA     300
Ind.  PEGPIMELNAKLWKAIAGSNGLIVNTFDAMEGRYVEHWNRDHRAGPRAWPIGPLCLAHGGTGTGTGAVEPSWMKWLDEKAAAGRAVLYVALGTAMAIPDA     300
                        1
Jap.  QLREVAGGLEAAAAAGVYFLWAVRPSDADLGAGFEERVEGRGMVVREWVDQWRILQHGCVRGFLSHCGWNSAVEGVAAGVPLAAWPMGAEQPLNAMLVVD     400
Ind.  QLREVAGGLEAAAAAGVDFLWAVRPSDADLGAGFEERVEGRGMVVREWVDQWRILQHGCVRGFLSHCGWNSAVEGVAAGVPLAAWPMGAEQPLNAMLVVD     400
                                                       2     3
Jap.  ELRVGVRVPVPTAMATGGHGVVGSEVIARVARELMMMAGEGKGGGGGEEARNVAALASKAREAVAEGGSSWKALEEMVATLCRPVEGDTPKPTK.          495
Ind.  ELRVGVRVPVPTAMATGGHGVVGSEVIARVARELMMMAGEGKGGGGGEAARNVASLASKAREAVAEGGSSWKALEEMVATLCRPVEGDTPKPTK.          495
```

Fig. S1. SNP haplotypes of LOC_Os04g24110 promoter, coding region, and protein sequences in *JAPONICA* and *INDICA* reference subspecies. (b) SNPs 1 and 4 in the LOC_Os04g24110 coding region were used for haplotype analysis in Fig. 1b-c. (c) Three non-synonymous amino acid changes resulting from three SNPs shown in (b) are highlighted with their corresponding numbers 1, 2, and 3.

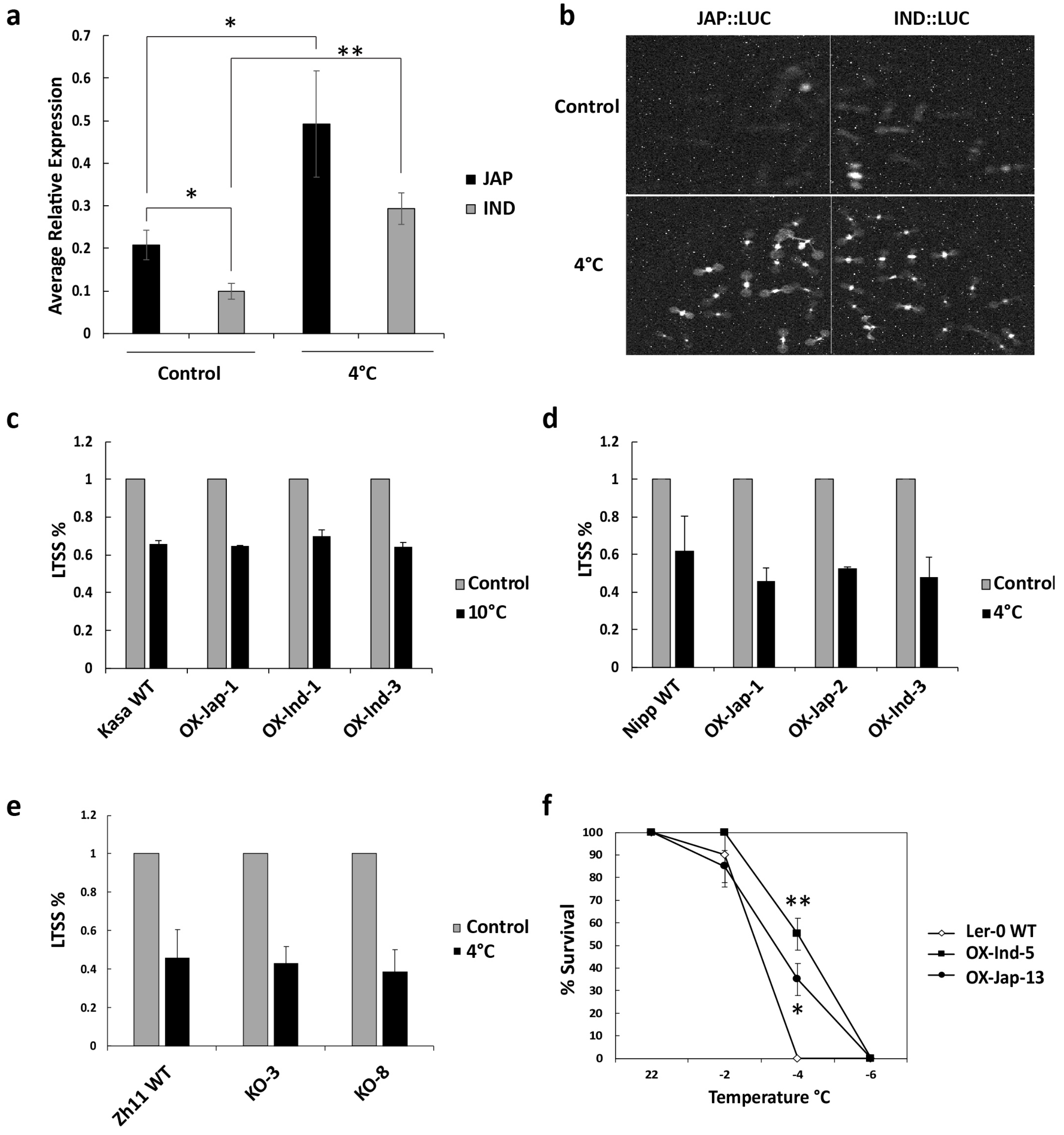

Fig. S2. ***In vivo OsUGT90A1*** **promoter activity in response to cold stress, and effect of** ***OsUGT90A1*** **overexpression (OX) or knockout (KO) on percent low temperature seedling survivability (LTSS%).** (a) Mean *OsUGT90A1* gene expression levels, determined by real-time qPCR analysis, in leaf tissues of three *JAPONICA* (JAP) accessions (H57-3-1, WIR 911, and M202) and three *INDICA* (IND) accessions (Djimoron, IR 238, and Sapundali Local) under 28°C/25°C control conditions and exposure to 4°C for 7 hrs. (b) *In vivo* real-time visualization of

luciferase bioluminescent signals in transgenic Arabidopsis under standard growth condition (control) and after chilling treatment (4°C) for 3 hrs. JAP::LUC: a 500 bp *OsUGT90A1* promoter fragment from the *JAPONCA* accession KRASNODARSKIJ 3352 fused to LUC; IND::LUC: a 500 bp *OsUGT90A1* promoter fragment from the *INDICA* accession CAROLINO 164 fused to LUC. (c-e) Effect of *OsUGT90A1* OX and KO on LTSS% in wild type (WT) Kasalath (Kasa), Nipponbare (Nipp), and Zhong Hua 11 (ZH11) or OX and KO transgenic rice plants. Ind and Jap indicate that the *INDICA* or *JAPONICA* allele of *OsUGT90A1*, respectively, was overexpressed. (f) Whole plant freezing survival phenotypes of wild type (WT) and *OsUGT90A1* OX transgenic Arabidopsis plants. Ind- and Jap- indicate that the *INDICA* or *JAPONICA* allele of *OsUGT90A1*, respectively, was overexpressed.

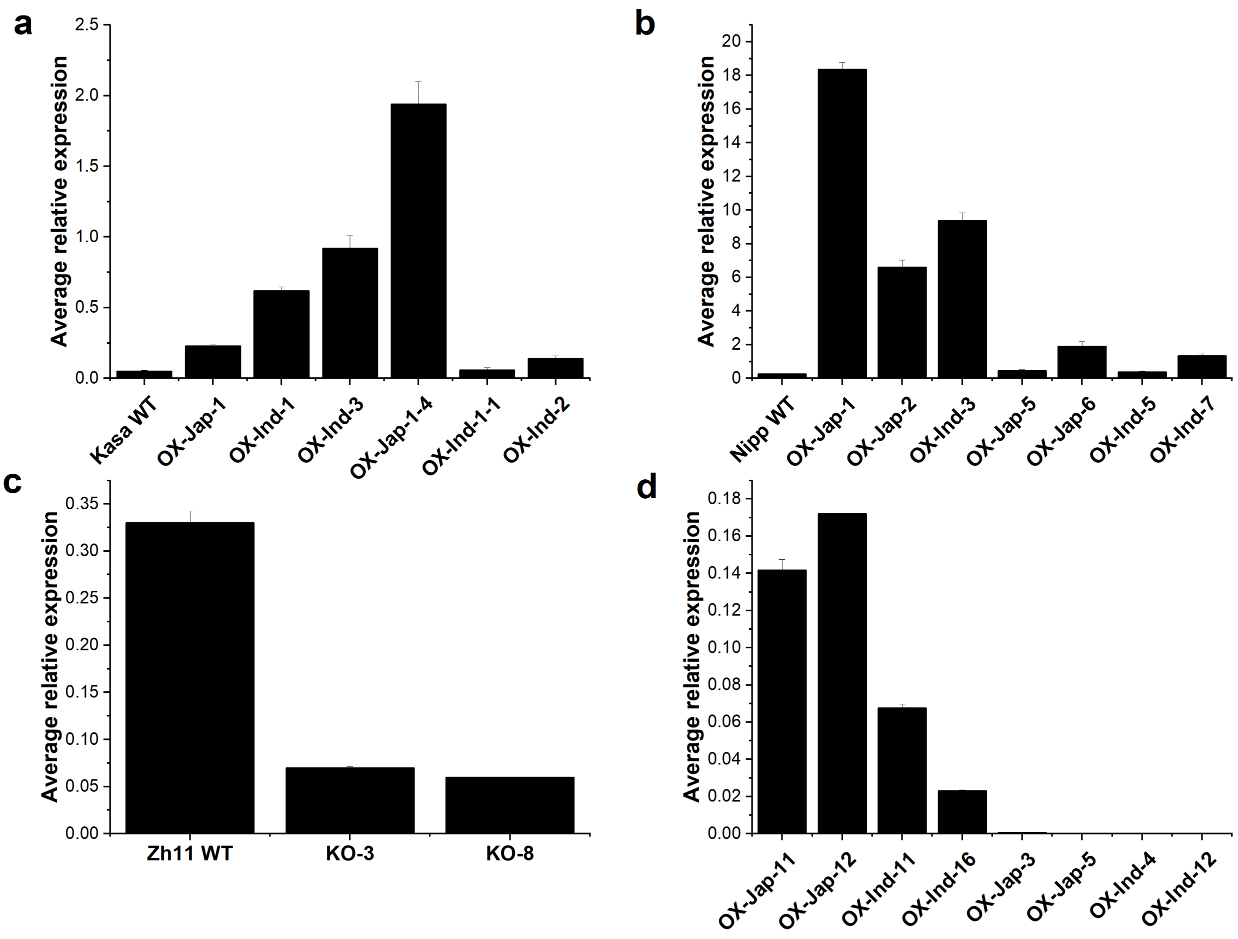

Fig. S3. Abundance of *UGT90A1* steady-state mRNA in individual transgenic rice and Arabidopsis plants used for analyses shown in Figs. 3-8, Fig. 10, and Fig. S4. The mean relative expression levels of *UGT90A1* in transgenic or wild type (except for heterologous Arabidopsis) plants are shown for: (a) Kasalath (Kasa) rice lines; (b) Nipponbare (Nipp) rice lines; (c) Zhong Hua 11 (Zh11) rice lines; and (d) Col-0 Arabidopsis lines.

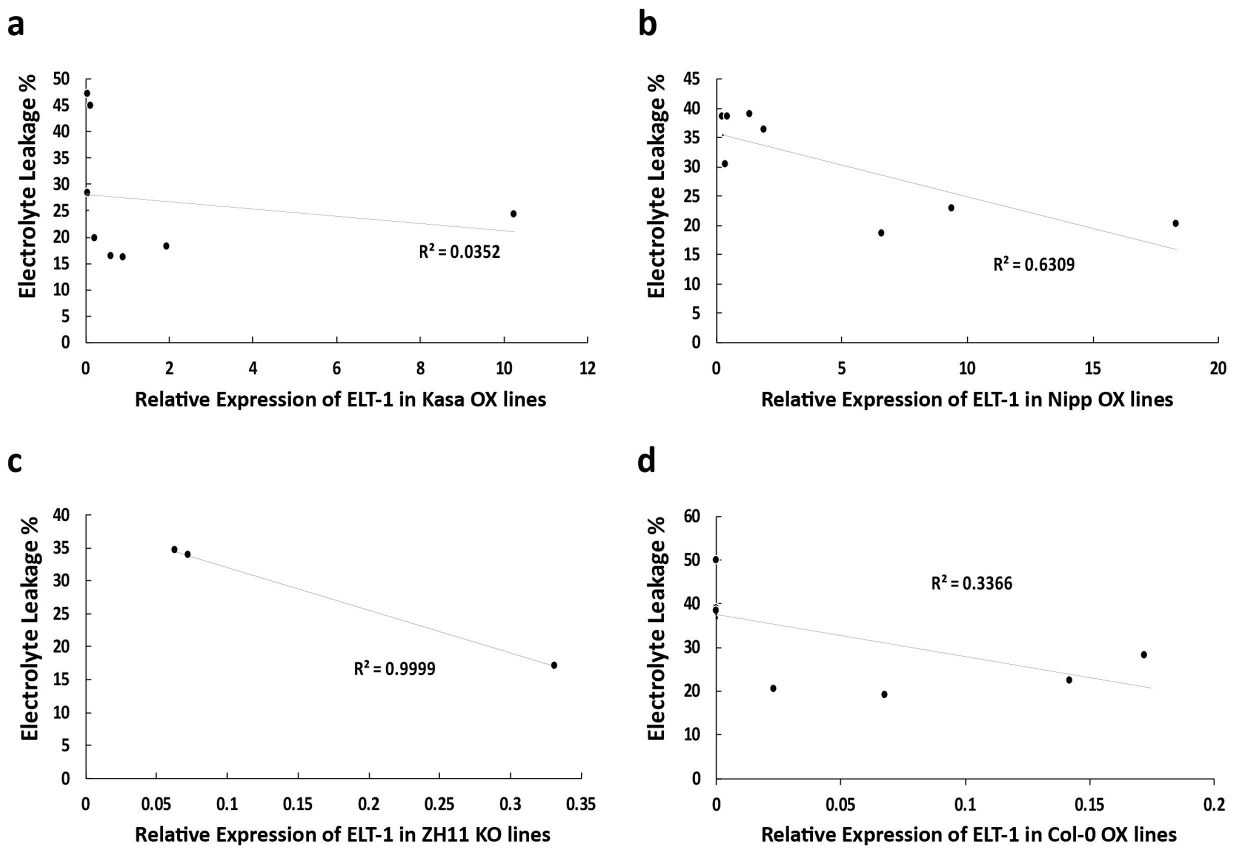

Fig. S4. Correlation of *OsUGT90A1* mRNA abundance with plasma membrane integrity in cold temperature stressed transgenic rice and Arabidopsis plants. Each dot represents an individual transgenic line in: (a) Kasalath (Kasa) rice; (b) Nipponbare (Nipp) rice; (c) Zhong Hua 11 (ZH11) rice; and (d) Col-0 Arabidopsis. Relative expression level of *OsUGT90A1* is shown on the x-axis and the corresponding median percent electrolyte leakage (%EL) as a measure of the degree of plasma membrane damage on the y-axis. Lines used in this figure are those both with significantly better than wild type %EL scores (Kasa: OX-Jap-1, Ind-1, Ind-3; Nipp: OX-Jap-1, Jap-2, Ind-3; Col-0: OX-Jap-11, Jap-12, Ind-11, Ind-16) and without significantly better than wild type %EL scores (Kasa: OX-Jap-1-4, Ind-1-1, Ind-2; Nipp: OX-Jap-5, Jap-6, Ind-5, Ind-7; ZH11: KO-3, KO-8; Col-0: OX-Jap-3, Jap-5, Ind-4, Ind-12).

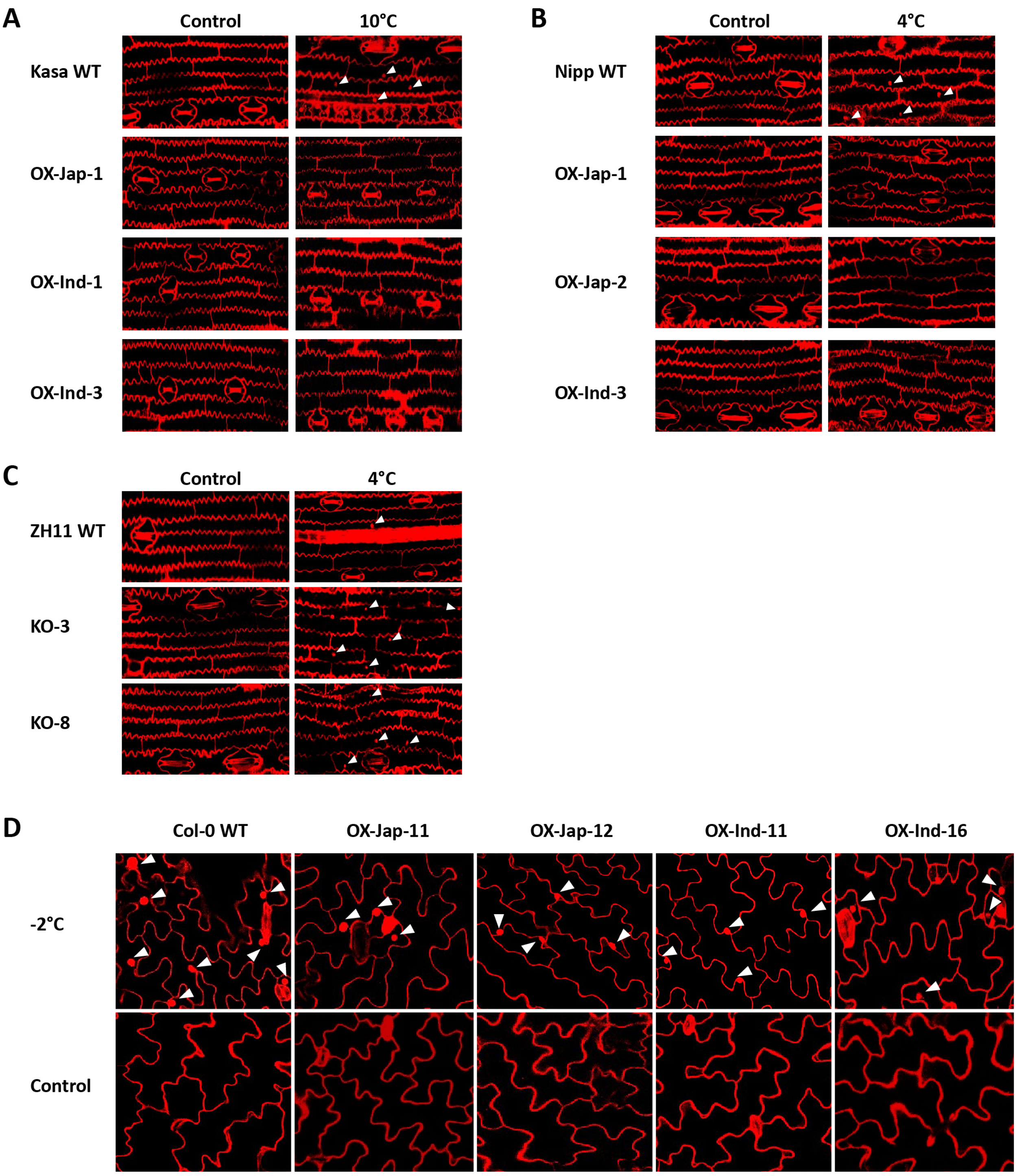

Fig. S5. Propidium iodide (PI) staining of wild type (WT) and *OsUGT90A1* overexpression (OX) and knockout (KO) transgenic rice and WT and OX transgenic Arabidopsis lines. (A-C) PI staining of the epidermal cell of leaf tissue in Kasalath (Kasa) OX, Nipponbare (Nipp) OX,

and in Zhong Hua 11 (ZH11) KO lines under normal growth conditions (control) or with chilling stress. (D) PI staining of the epidermal cell of the leaf tissue in Col-0 OX lines under normal growth conditions (control) or with mild freezing stress. Damaged cells with PI-stained red nucleus are labeled by arrows. Ind and Jap indicate that the *INDICA* or *JAPONICA* allele of *OsUGT90A1*, respectively, was overexpressed.

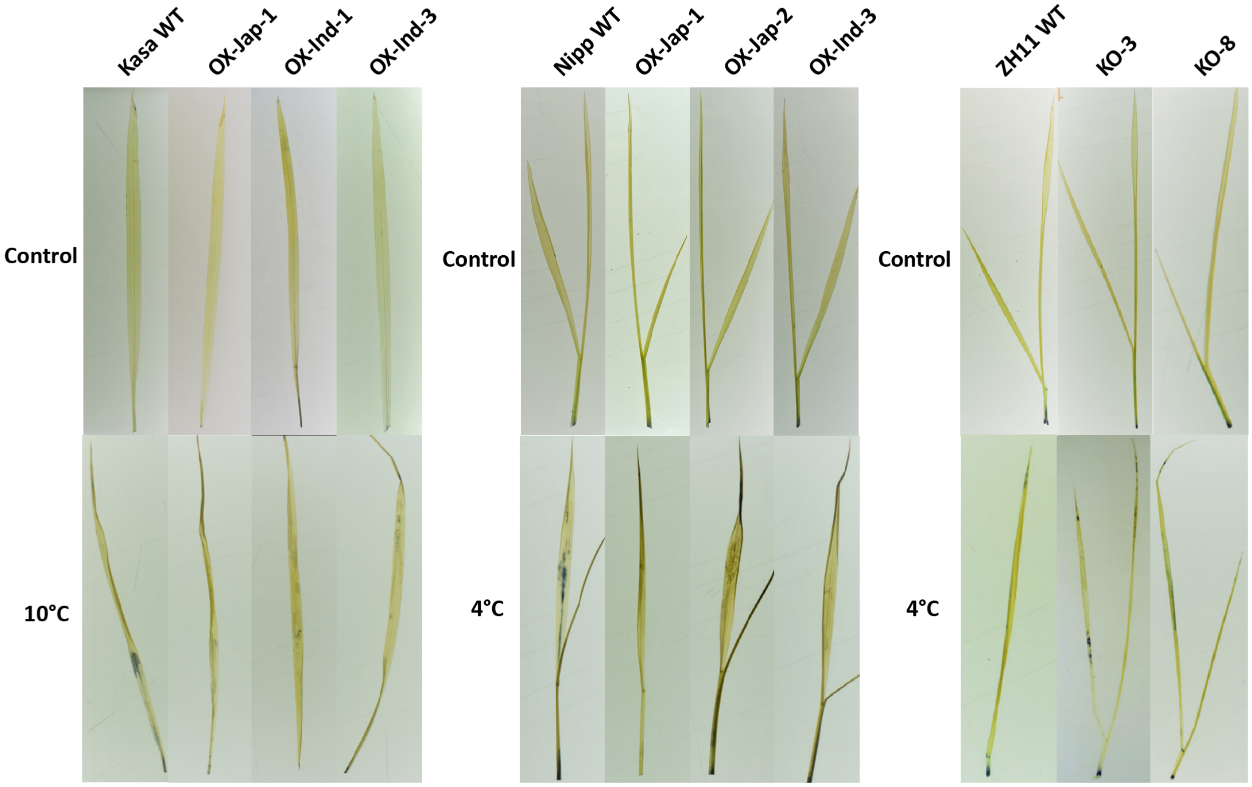

Fig. S6. Effect of *OsUGT90A1* overexpression (OX) and knockout (KO) on ROS scavenging activity under standard growth (control) or chilling stress conditions in transgenic rice. Nitro Tetrazolium Blue staining (blue) was used to qualitatively visualize the ROS $^{\bullet}O_2^{-}$ in leaves of wild type (WT) and OX lines in the Kasalath (Kasa; left) or Nipponbare (Nipp; middle) background, and of WT and KO lines in the Zhong Hua 11(ZH11; right) background. Ind and Jap indicate that the *INDICA* or *JAPONICA* allele of *OsUGT90A1*, respectively, was overexpressed.

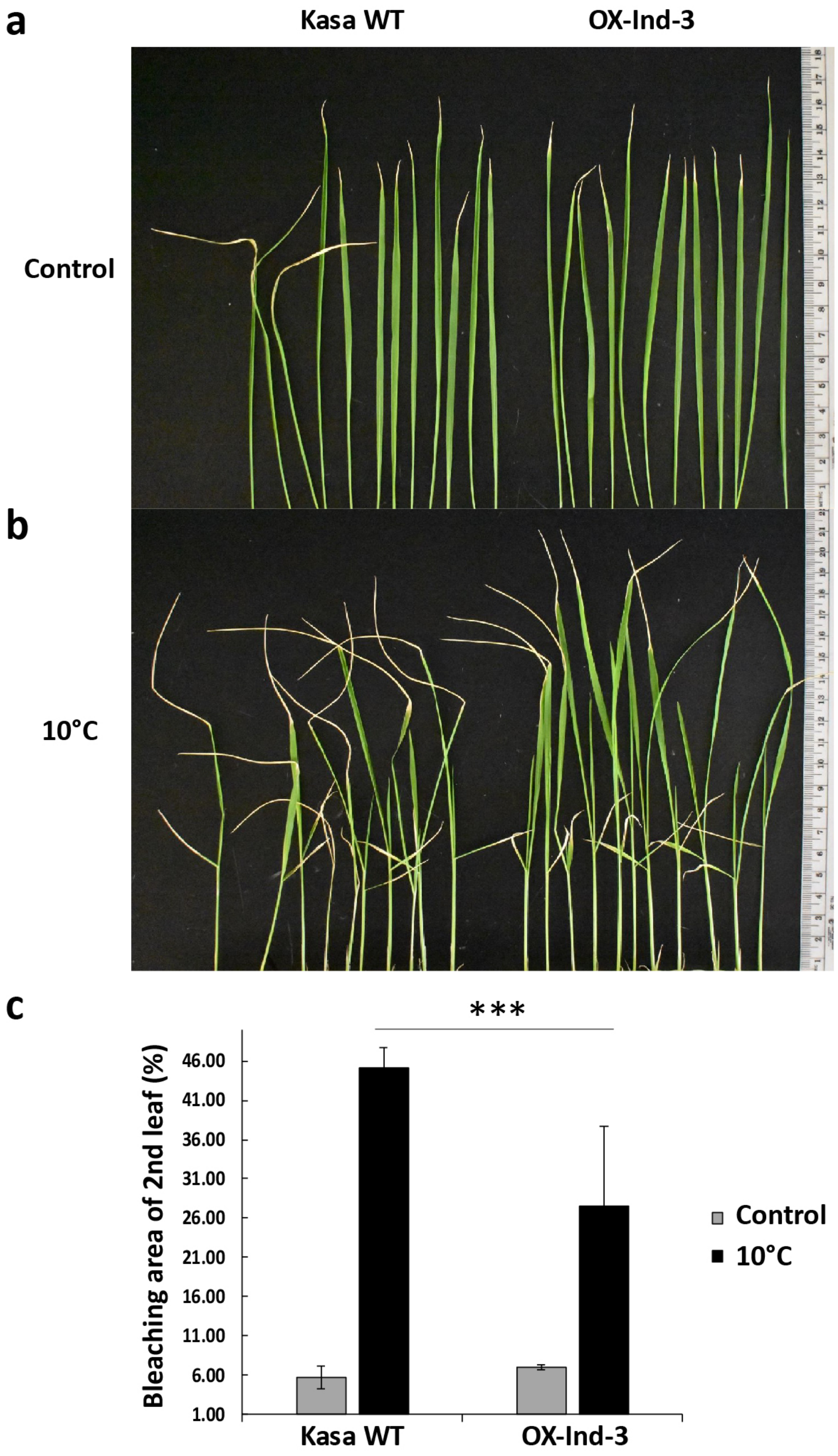
a
Kasa WT
OX-Ind-3
Control
b
10°C
c
***
Bleaching area of 2nd leaf (%)
46.00
41.00
36.00
31.00
26.00
21.00
16.00
11.00
6.00
1.00
Kasa WT
OX-Ind-3
Control
10°C

Fig. S7. Leaf bleaching (necrosis) phenotype of wild type (WT) Kasalath (Kasa) and *OsUGT90A1* overexpression (OX) Kasa lines, and quantification of necrotic sectors. (a) Leaf tip bleaching phenotype in the second leaf of 23-day-old seedlings grown under standard growth conditions (control). (b) Leaf tip bleaching phenotype in the second leaf of 23-day-old seedlings exposed to 10°C for two days and after one week of recovery growth. (c) Quantification of the median percent (%) bleached area of the 2nd leaves shown in (a) and (b). Ind indicates that the *INDICA* allele of *OsUGT90A1* was overexpressed. ***: P <0.001 (two-tailed Student's t-test).

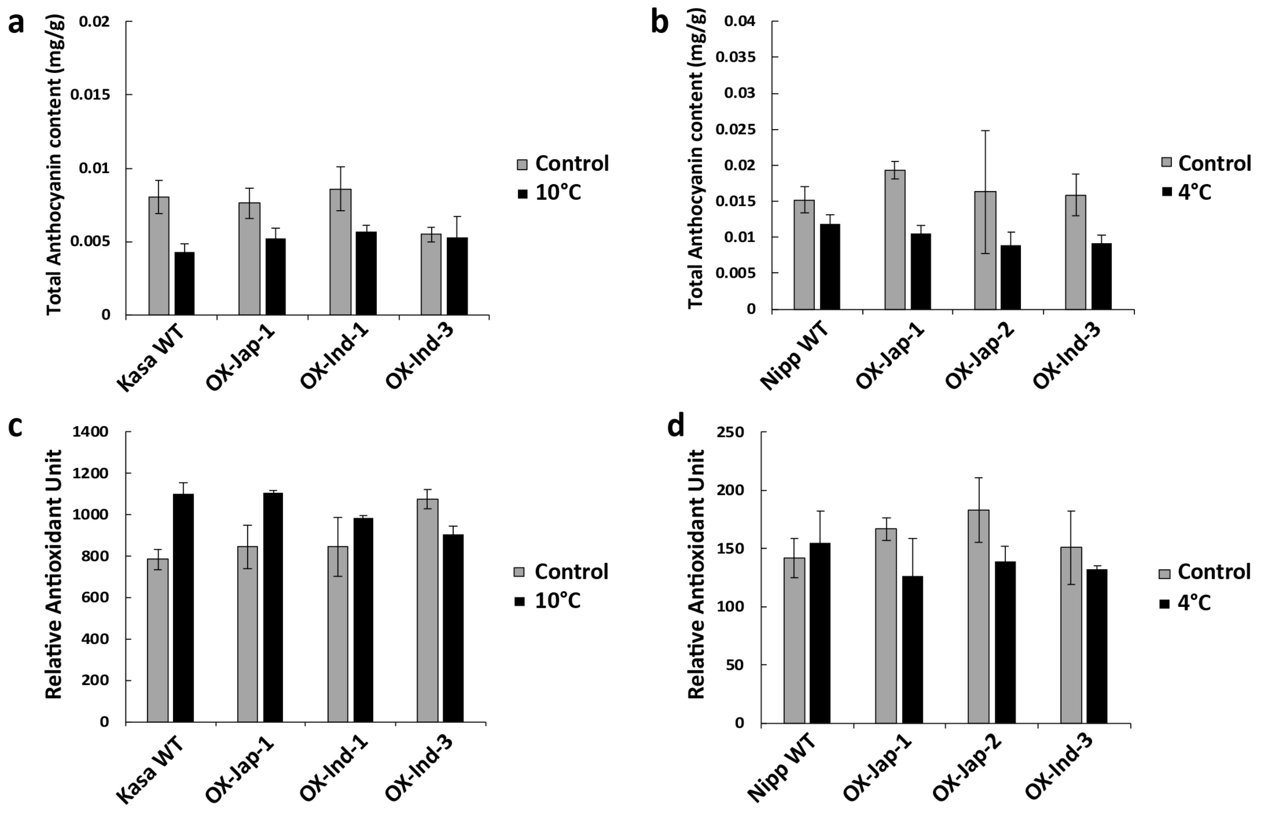

Fig. S8. Overall concentration and non-enzymatic antioxidant activity of total flavonoid/anthocyanin content extracted from leaf tissue of wild type (WT) and *OsUGT90A1* overexpression (OX) transgenic rice plants. (a) and (b), total flavonoid/anthocyanin content in leaf tissue of Kasalath (Kasa) and Nipponbare (Nipp) OX lines, respectively. (c) and (d), non-enzymatic antioxidant activity of flavonoid/anthocyanin extracts shown in (a) and (b) determined by the 2,2-diphenyl-1-picrylhydrazyl (DPPH) reduction assay.

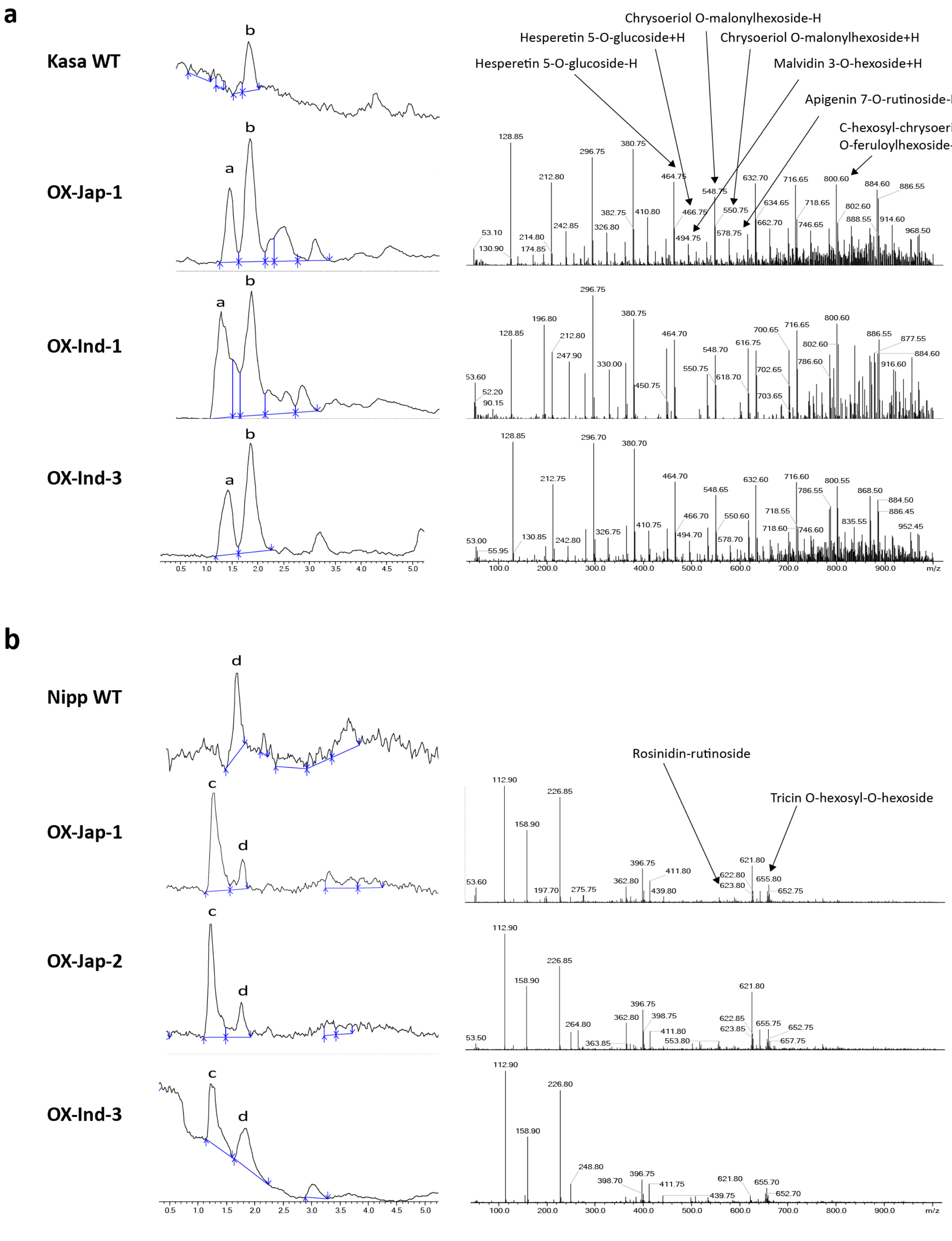

Fig. S9. Effect of *OsUGT90A1* overexpression (OX) on flavonoid/anthocyanin composition under chilling stress. (a) and (b), HPLC-MS assay determined specific flavonoid/anthocyanin

composition groups (left) and compounds (right) in wild type (WT) or *OsUGT90A1* OX Kasalath (Kasa) and Nipponbare (Nipp) lines, respectively.

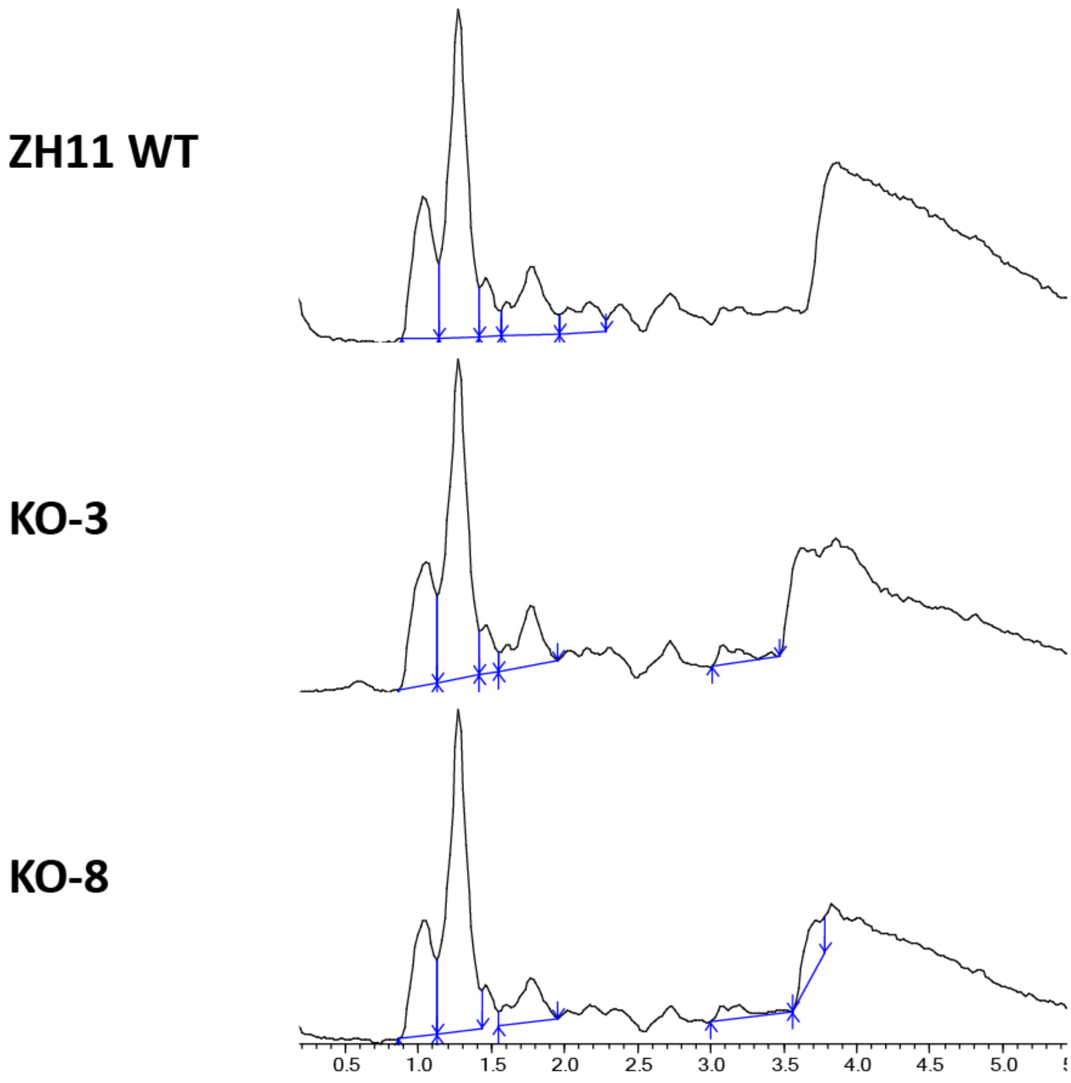

Fig. S10. Effect of *OsUGT90A1* knockout (KO) on flavonoid/anthocyanin composition under chilling stress. HPLC-MS assay determined specific flavonoid/anthocyanin composition groups in *elt1* KO mutants in the ZH11 background.

Table S1. Primer information

Gene	Forward (F) and reverse (R) primers	Purpose
OsUGT90A1	F: AGATCTACTTATTTTGTGGGTTTCGGTG<br>R: GAATTCGCTCCAGGCCGGTCATG	Overexpression construct
OsUGT90A1	F: GGTACCGAGGAAACAGCACCCGCATAC<br>R: GGATCCTGACCGGCCTGGAGCTAGC	promoter::LUC fusion
OsUGT90A1	F: CTCGAGCTCCACTGCTAGCTCCAG<br>R: AGATCTTTTTGTGGGTTTCGGTGTATCTCCTTC	eGFP fusion protein
OsUGT90A1	GGCGACGACATGGTCCGGGA	Knockout construct
OsUGT90A1	F: AGATCTATGGCGGCGGCTGGTCA<br>R: GAATTCACTTATTTTGTGGGTTTCGGTG	qRT-PCR
OsUGT90A1	F: ACCACTTCGACCGCCACTACT<br>R: ACGCCTAAGCCTGCTGGTT	qRT-PCR
OsUBQ	F: ACCACTTCGACCGCCACTACT<br>R: ACGCCTAAGCCTGCTGGTT	qRT-PCR
